# Heme Oxygenase Dependent Bilirubin Generation in Vascular Cells: A Role in Preventing Endothelial Dysfunction in Local Tissue Microenvironment?

**DOI:** 10.3389/fphys.2020.00023

**Published:** 2020-01-29

**Authors:** Mariapaola Nitti, Anna Lisa Furfaro, Giovanni E. Mann

**Affiliations:** ^1^Department of Experimental Medicine, University of Genoa, Genoa, Italy; ^2^King’s British Heart Foundation Centre of Research Excellence, School of Cardiovascular Medicine & Sciences, Faculty of Life Sciences & Medicine, King’s College London, London, United Kingdom

**Keywords:** HMOX1, bilirubin, endothelial cells, diabetes, atherosclerosis, hypertension, obesity, tumor

## Abstract

Among antioxidants in the human body, bilirubin has been recognized over the past 20 years to afford protection against different chronic conditions, including inflammation and cardiovascular disease. Moderate increases in plasma concentration and cellular bilirubin generation from metabolism of heme via heme oxygenase (HMOX) in virtually all tissues can modulate endothelial and vascular function and exert antioxidant and anti-inflammatory roles. This review aims to provide an up-to-date and critical overview of the molecular mechanisms by which bilirubin derived from plasma or from HMOX1 activation in vascular cells affects endothelial function. Understanding the molecular actions of bilirubin may critically improve the management not only of key cardiovascular diseases, but also provide insights into a broad spectrum of pathologies driven by endothelial dysfunction. In this context, therapeutic interventions aimed at mildly increasing serum bilirubin as well as bilirubin generated endogenously by endothelial HMOX1 should be considered.

## Introduction

Bilirubin is widely known as an end-product of the degradation of heme which occurs in the spleen primarily as part of red blood cell removal. Senescent or damaged red blood cells are degraded and the heme groups derived from hemoglobin are converted into biliverdin by the activity of heme oxygenases (HMOX1 and HMOX2) (Maines, 1988), and then converted into bilirubin via the activity of BLVRA. This form of bilirubin, known as UCB, is bound to albumin to enable its plasma transport to the liver where it is conjugated with glucuronic acid and excreted as main component of bile (Kapitulnik, 2004).

The elevation of bilirubin concentration in plasma is well known as a marker of hemolytic conditions, liver damage or bile-duct impairment. When the concentration of UCB exceeds the binding capability of albumin, the fraction of free bilirubin (unbound UCB) increases and can cause toxicity in the nervous system in particular, a serious condition known as bilirubin encephalopathy which has been the focus of excellent reviews (Ostrow et al., 2003; Shapiro, 2003; Kapitulnik, 2004). It is important to note that neuronal cells are particularly sensitive to UCB toxicity compared to other cell types (Silva et al., 2002). Indeed, neuronal toxicity is observed *in vitro* even at relatively low concentrations, e.g., 0.5 μM UCB is toxic to primary cells and acts to enhance glutamate-induced toxicity (Grojean et al., 2000). Moreover, mitochondrial damage is critically involved in neuronal damage induced by UCB especially in developing neurons (Rodrigues et al., 2002).

Nonetheless, different beneficial aspects linked to mild elevations of plasma bilirubin (up to 12 mg/dl) have been recognized. The anti-inflammatory effect of jaundice was already observed by Hench (1938) in patients affected by rheumatoid arthritis and by Engerman and Meyer (1959) in hyperbilirubinemic rats. More recent evidence comes from individuals with GS, where bilirubin affords cardiovascular protection (Vítek et al., 2002; Novotný and Vítek, 2003; Maruhashi et al., 2012; Gazzin et al., 2016). Furthermore, the degradation pathway of heme occurs in virtually all tissues, since heme-containing proteins are ubiquitously distributed and undergo physiological turnover. Notably, also the generation of bilirubin in peripheral tissues has been proposed to be protective (Sedlak et al., 2009; Takeda et al., 2015; Nam et al., 2018), and bilirubin derived from vascular cells could modulate tissue microenvironment playing both antioxidant and anti-inflammatory roles (Siow et al., 1999; He et al., 2015). We pointed out this aspect in the different contexts analyzed in the present review. Importantly, studies of cytoprotective pathways in endothelial cells is receiving considerable attention in view of potential therapeutic targets to maintain vascular health or to delay or treat vasculopathies (Kim Y.-M. et al., 2011; Calay and Mason, 2014; Mason, 2016).

## Biosynthesis, Metabolism, Bioavailability and Activity

Heme oxygenase (HMOX) is the first, rate-limiting enzyme in heme degradation pathway, with two major isoforms of HMOX identified. HMOX1 is inducible, expressed only under oxidative stress or when heme concentration increases; HMOX2 is constitutively present mainly in testis and neuronal cells (Maines et al., 1986) but has also been detected in vascular cells (Chen et al., 2014). HMOX catalyzes the opening of the prothoporphyrinic ring of heme, generating biliverdin, free iron (Fe^2+^) and CO (Maines, 1988). The activity of BLVRA converts biliverdin into UCB. Heme degradation occurs in all cell types in order to complete the turnover of heme-containing proteins. Furthermore, HMOX1 induction crucially drives cell adaptive responses to stressors and its metabolic products exert potent biological activities (Siow et al., 1999; Otterbein et al., 2016; Kishimoto et al., 2019). CO has anti-apoptotic and anti-inflammatory activities (Ryter et al., 2002), both intracellularly and in the microenvironment (Morita and Kourembanas, 1995) of blood vessels by increasing cGMP concentration and activating the MAPK pathway (Siow et al., 1999; Ryter et al., 2002). The generation of free iron is potentially highly toxic but, under physiological conditions, a parallel induction of the heavy chain of ferritin, and the activation of membrane Fe-ATPase transporters occurs (Dulak et al., 2002; Loboda et al., 2008). This adaptation is critical in decreasing intracellular Fe^2+^ content and prevents the generation of ROS via the Fenton reaction, and thereby maintains endothelial function (Balla et al., 2007). Finally, bilirubin is a potent antioxidant, as highlighted by the pioneering studies of Stocker et al. (1987). It has been well demonstrated that bilirubin efficiently scavenges ROS, peroxynitrite and peroxyradicals (Neuzil and Stocker, 1993). Moreover, as it is lipophilic, bilirubin is able to prevent lipid peroxidation (Stocker et al., 1987). Indeed, in HEK293 cells, the ability of UCB to prevent lipid peroxidation has been well documented (Sedlak et al., 2009), e.g., in hepatoblastoma cells and plasma samples exposed to oxidative stressors and LPS (Zelenka et al., 2012). Notably, UCB was shown to prevent LDL oxidation, 20 times more efficiently than vitamin E (Wu et al., 1994).

**FIGURE 1:**
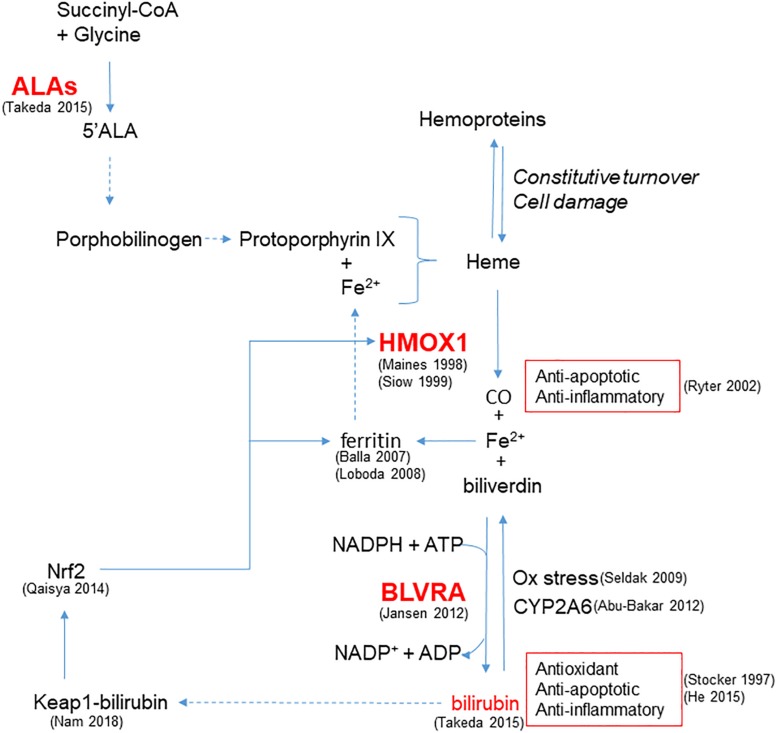
Enzymatic reactions modulated to increase bilirubin generation and cytoprotection. HMOX1 catalyzes the degradation of heme groups to CO, Fe^2+^ and biliverdin, the latter subsequently converted to bilirubin by BLVRA. By reaction with oxidant species, bilirubin is oxidized back to biliverdin, amplifying the antioxidant effect. Bilirubin is also the substrate of CYP2A6 responsible for its oxidation to biliverdin. Bilirubin and CO exert anti-apoptotic and anti-inflammatory activity. Fe^2+^ is quenched by the heavy chain of ferritin, and further released to form heme. In addition to iron availability, the synthesis of heme groups depends on the activity of ALAs that catalyzes the reaction between succinyl-CoA and glycine to form 5’ALA; this is then converted to porphobilinogen and protoporphyrin IX that forms heme. Heme groups can also derived from the constitutive turnover of hemoproteins that can be amplified by cell damage. A positive feedback of cytoprotection can be generated by the ability of bilirubin to bind nucleophiles such as thiol reactive cysteines on Keap1, favoring Nrf2-dependent HMOX1 gene transcription. References in brackets.

In addition to HMOX1, modulation of other steps involved in bilirubin production, from the synthesis of heme rings to the reduction of biliverdin catalyzed by BLVRA, plays a role in cellular protection (Figure 1). Indeed, recent evidence from different cell lines suggests that a continuous *de novo* synthesis of heme occurs with the specific purpose of bilirubin generation in order to provide cytoprotection (Takeda et al., 2015). Indeed, by inhibiting the synthesis of ALA the synthesis of bilirubin is reduced, leading to increased cell damage in response to oxidative stress and demonstrating that endogenous bilirubin is highly efficient in preventing cell damage.

Furthermore, BLVRA contributes to the antioxidant properties of the HMOX1/bilirubin system, as pointed out by Jansen and Daiber (2012). Importantly, Nam et al. (2018) have recently discussed the physiological relevance of the conversion of biliverdin to bilirubin through an energy consuming reaction, highlighting that the reaction carried out by BLVRA dramatically increases the electrophilicity of bilirubin in comparison to biliverdin. This enables its binding to thiol reactive cysteines on Keap1, favoring Nrf2-dependent gene induction and thereby generating a positive feedback of cytoprotection. Moreover, the up-regulation of Nrf2 induced by bilirubin treatment has already been demonstrated in neuronal-like cells (Qaisiya et al., 2014) and is of relevance in the context of maintaining vascular health. Notably, bilirubin can be oxidized back to biliverdin and then be regenerated by the activity of BLVRA. The biliverdin-bilirubin cycle has been postulated to be highly active under reducing oxidative stress (Baranano et al., 2002) and, by cooperating with GSH (Sedlak et al., 2009), is able to counteract a 10,000-fold excess of H_2_O_2_ (Doré et al., 1999), affording protection in endothelial cells (Jansen et al., 2010). Moreover, mitochondrial CYP2A6 is able to perform the same oxidation of UCB back to biliverdin, highlighting the biological importance of biliverdin-bilirubin redox cycle to maintain cell homeostasis (Abu-Bakar et al., 2012).

UCB is able to bind different proteins to facilitate crossing plasma or intracellular membranes. As widely reviewed (Bellarosa et al., 2009), it can bind ABC transporters (ABCB1, ABCC1,2,3, ABCG2 and the OATP). In plasma, UCB binds to albumin and to enable uptake in the liver, it binds to transport proteins initially identified as ligandins (Litwack et al., 1971) and subsequently recognized as members of GSH transferases (Habig et al., 1974). Especially in the liver, UCB is transformed to conjugated bilirubin by the activity of uridine diphospho-glucoronosyl transferase 1A1 (UGT1A1) in order to increase its water solubility. Hepatic bilirubin metabolism and transport have been widely investigated (Kamisako et al., 2000).

Interestingly, as far as the intracellular transport is concerned, it was postulated as early as 1969 (Levi et al., 1969) that UCB could bind FABPs which are involved in the intracellular transport of organic anions. In this context, the discovery of UnaG protein from freshwater eel (Kumagai et al., 2013), a protein belonging to FABPs that selectively binds bilirubin and generates a fluorescent signal, improved the detection of endogenously generated bilirubin enabling studies of its subcellular localization and transport. Endogenous UCB seems to be mainly located at ER cytosolic membranes, associated with HMOX1/BLVRA activities and has also been detected in the nucleus and mitochondria (Park et al., 2016). However, intracellular transport mechanisms of endogenous UCB and its role in subcellular compartments remain to be explored.

Among the different cell types, neuronal cells are highly sensitive to UCB toxicity especially during development, and undergo apoptosis at relatively low concentrations (0.25–5 μM) of UCB (Grojean et al., 2000; Rodrigues et al., 2002). In addition, as far as endothelial cells are concerned, different sensitivities to UCB have been postulated depending on the different tissues from which endothelial cells were derived. Moreover, immortalized endothelial cell lines, such as bEnd.3 cells used as a model of BBB, undergo oxidative stress and apoptosis when treated with 20–40 μM UCB, while no significant damage occurs in endothelial cells derived from pancreatic islets of Langerhans (Kapitulnik et al., 2012). The peculiar sensitivity of BBB-like endothelial cells has been confirmed by other authors (Palmela et al., 2012), underlining the marked sensitivity of brain endothelial cells and neurons to UCB, as previously reviewed (Ostrow et al., 2003; Brito et al., 2014).

However, regardless of the endothelial cell type, the protective, pro-survival activity of low concentrations of UCB (0.1–5 μM) has been widely documented and ascribed to its antioxidant properties (Kapitulnik et al., 2012). Thus, although the different cell types exhibit varying degrees of sensitivity toward bilirubin toxicity, they all benefit from bilirubin cytoprotection at low concentrations. This has clinical implications, since recent research has shown that BRT prevents ROS generation in *ex vivo* platelets, opening the possibility of screening the actions of acute BRT treatment to improve platelets storage and function (Pennell et al., 2019).

## Effects on Vascular Redox Status: Implications for Hypertension, Diabetes and Ischemia/Reperfusion Injury

The molecular mechanisms underlying the antioxidant activity of UCB in endothelial cells have been investigated extensively in the context of various pathophysiological conditions and diseases. Cytoprotection afforded by UCB has been attributed to quenching of superoxide anions derived from NOXs (Datla et al., 2007). Indeed, HMOX1 induction by hemin prevents NOX2/4 activation in apolipoprotein E-deficient mice characterized by vascular oxidative stress, and the same result is observed following bilirubin treatment *in vitro* or HMOX1 overexpression via gene transfer in VSMC (Datla et al., 2007). Notably, UCB derived from HMOX1 in endothelial cells has been shown to be able to scavenge NADPH oxidase derived ROS, impairing leukocyte transmigration and inflammatory responses (Vogel and Zucker, 2016), as discussed in Section “Effects on Adhesion Molecule Expression: Implications for Obesity, Atherosclerosis and Chronic Inflammatory Diseases.”

Protection afforded by bilirubin in endothelial cells has recently been reported Ziberna et al. (2016), who demonstrated that HMOX1 induction in response to CoPPIX or cell supplementation with a very low concentration of bilirubin (≤50 nM) significantly elevates intracellular bilirubin levels, leading to increased antioxidant defenses. It is important to note that bilirubin derived from HMOX2 can also protect vascular cells. Notably, based on the co-localization of HMOX2 with NOX4 in cerebral microvascular endothelial cells, it has been hypothesized that bilirubin may scavenge ROS derived from TNFα induced NOX4 activation (Basuroy et al., 2009). Thus, there is accumulating evidence that low concentration of UCB, both plasma and endothelial cell derived, can affect endothelial redox balance and thereby attenuate cell damage in oxidative stress.

### Bilirubin and Hypertension

Slight increases in plasma total bilirubin concentrations (1.53 ± 0.48 mg/dl) have been reported to preserve flow-mediated vasodilation compared to subjects with low levels of plasma bilirubin (0.40 ± 0.08 mg/dl) (Erdogan et al., 2006). As recently reviewed (Mortada, 2017), a negative correlation between serum level of bilirubin levels and hypertension has been established in different studies (Mortada, 2017). The antihypertensive activity of a moderate hyperbilirubinemia is related to an increase in NO bioavailability due to the reduction of AngII-dependent superoxide generation by endothelial cells (Pflueger et al., 2005; Vera et al., 2009). Indeed, bilirubin quenches superoxide anion derived from NOX in cultured vascular endothelial cells exposed to AngII and increases the bioavailability of NO, leading to vascular relaxation and lowering of blood pressure (Pflueger et al., 2005; Fujii et al., 2010).

**FIGURE 2:**
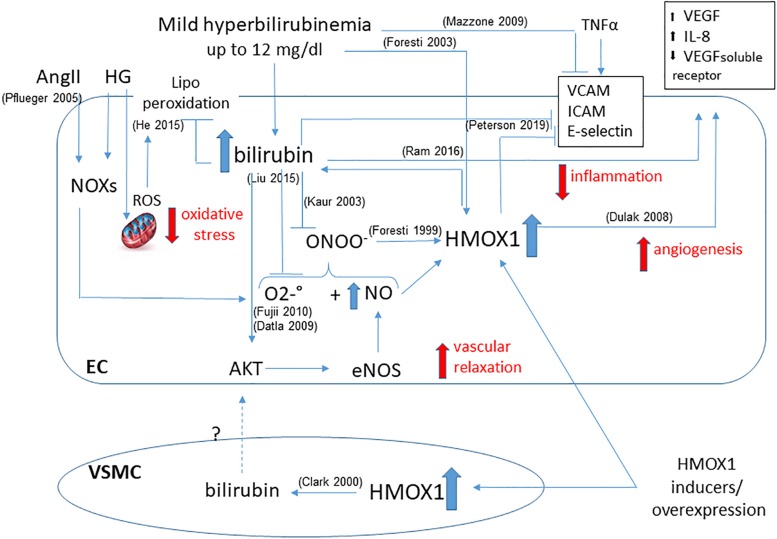
Functional effects of bilirubin on endothelial cells. Positive loop between plasma- and HMOX1 derived bilirubin is shown highlighting the main activities in maintaining endothelial function. The ability to reduce oxidative stress by quenching both O_2_^–.^ and ONOO^–^ prevents the dysfunction due to HG or AngII. The increased bioavailability of NO favors vascular relaxation and further activates HMOX1. The direct activity of bilirubin and HMOX1 in preventing the expression of adhesion molecules (VCAM, ICAM, E-selectin) is showed as well as the generation of angiogenetic factors (VEGF, IL-8, VEGF soluble receptor). A further contribution from VSMC-derived bilirubin has been hypothesized. References in brackets.

Furthermore, HMOX1 derived UCB protects endothelial cells against nitrosative stress, induced by hemin pre-treatment, which is able to further increase HMOX1 dependent bilirubin generation to reduce endothelial apoptosis induced by peroxynitrite (Foresti et al., 1999). Indeed, NO and peroxynitrite are able to induce HMOX1 in endothelial cells, and bilirubin may directly scavenge NO to prevent excessive accumulation (Kaur et al., 2003) and the formation of peroxynitrite-dependent protein modification in human plasma (Minetti et al., 1998). Thus, a positive loop of cytoprotection seems to exist between NO and HMOX1-derived bilirubin (Figure 2).

Moreover, the increased availability of NO due to UCB, positively affects the rate of glomerular filtration improving renal hemodynamics (Vera and Stec, 2010). However, it has also been hypothesized that other mechanisms in addition to superoxide scavenging can be involved in the ability of bilirubin to lower blood pressure and decrease hypertension, for instance the modulation of calcium and potassium channels (Stec et al., 2013). In fact, it has been shown that the induction of HMOX1, through the generation of bilirubin, favors vessel relaxation by acting on the transport of calcium and potassium, and thereby improving EDH in a rat model of spontaneous hypertension (Li et al., 2013).

### Bilirubin and Diabetes

The role played by UCB in preventing endothelial dysfunction in chronic sequaele of diabetes has been widely demonstrated (Vítek, 2012). Indeed, the incidence of retinopathy, coronary artery disease, and cerebrovascular diseases is low in Gilbert subjects (i.e., bilirubin level 1.2 mg/dl) with diabetes (Inoguchi et al., 2007). Moreover, the Hisayama Study revealed that increased bilirubin levels counteract diabetic retinopathy (Yasuda et al., 2011), with total bilirubin concentration negatively correlated with the severity of retinopathy (Sekioka et al., 2015).

Furthermore, in diabetic rats, up-regulation of HMOX1 increases serum bilirubin, reduces superoxide anion and endothelial sloughing induced by hyperglycemia (Quan et al., 2004). Moreover, we have reported that activation of the HMOX1/bilirubin pathway protects endothelial cells against HG-induced damage by reducing HNE production and lipid peroxidation (He et al., 2015). In addition, as mentioned before, bilirubin acts by inhibiting NOX. This action, at the level of endothelial cells and mesangial cells in diabetic kidney, has been shown to ameliorate diabetic nephropathy (Fujii et al., 2010). An elegant study by Liu et al. (2015), in different animal models of diabetes, established that HMOX1 overexpression improves vascular function, but that BLVRA activity is also required, underlining that conversion of biliverdin into bilirubin is fundamental. These authors also established that administration of exogenous bilirubin affords vascular protection by restoring AKT-dependent signal transduction (Liu et al., 2015). AKT seems to be a key target in bilirubin activity in endothelial cells, and the ability to restore the AKT-eNOS pathway is involved in not only maintaining endothelial redox balance and cell survival but also pro-angiogenic signals (see section “Effects on Angiogenesis: Implications for Wound Healing, Vascular Dysfunction in Pregnancy and Tumor Growth”). In addition, in a mouse model of obesity, bilirubin has been shown to activate AKT, reducing insulin resistance in skeletal muscle (Dong et al., 2014) and improving glucose tolerance. The role of HMOX1 and BLVRA in inflammation associated with diabetes has recently been reviewed (Rochette et al., 2018).

### Bilirubin and Ischemia/Reperfusion Injury

Bilirubin has been shown to be protective against the development of ischemic damage associated with ischemic/reperfusion injury and endothelial activation (Yang et al., 2016). A recent cross-sectional study performed on 1121 healthy Japanese demonstrated that low levels of plasma bilirubin are associated with a high prevalence of ischemic modifications in brain white matter (Higuchi et al., 2018). In this context, a recent review form Thakkar et al. (2019) rigorously analyzed clinical studies highlighting a relationship between bilirubin levels and cerebral ischemic stroke. These authors provided evidence that in the large majority of the studies, a negative relationship exists between the level of serum bilirubin and prevalence of stroke. However, they also discussed limitations of some of these studies that only considered total bilirubin levels and ischemic stroke without considering hemorrhagic stroke or traumatic brain injury. Interestingly, recent evidence in a rat model of MCAO established that intraperitoneal administration of biliverdin significantly reduces the infarct size by modulating microRNA-mRNA network (Zou et al., 2019). It is important to note that at the level of the nervous system, up-regulation of HMOX1 can be both protective and damaging as reviewed recently (Nitti et al., 2018), but the role played by HMOX1 derived bilirubin in neurons and non-neuronal cells in neuroprotection has been highlighted (Hung et al., 2010).

In different models of cardiac ischemia/reperfusion damage, bilirubin administration protects against tissue damage. Indeed, *ex vivo* heart perfusion with BRT improves post-ischemic outcomes by reducing oxidative damage (Bakrania et al., 2016). Moreover, intraperitoneal administration of bilirubin has been shown to prevent cardiolipin oxidation and to reduce the infarct size in a rat model of coronary ischemia/reperfusion injury (Ben-Amotz et al., 2014). More recently, preconditioning performed using nanoparticles of pegylated bilirubin was highly effective in preventing hepatic ischemic damage (Kim et al., 2017). Furthermore, the critical importance of the endogenous HMOX1/bilirubin pathway has been demonstrated following supplementation cell culture media not only with UCB but also with hemin. Both reduce ROS generation induced by re-oxygenation of cardiomyocytes subjected to hypoxia, preventing cell injury (Foresti et al., 2001). Furthermore, hemin efficiently prevents oxidative damage in VSMCs by increasing bilirubin generation (Clark et al., 2000). Even though the majority studies performed *in vivo* or *ex vivo* evaluate whole tissue without considering the specific role played by endothelial cells, it is conceivable that bilirubin supplementation can directly protect the endothelium. It is important to note that UCB induces HMOX1 up-regulation in aortic endothelial cells, generating a positive loop that increases endothelial resistance to oxidative stress (Foresti et al., 2003). The specific anti-inflammatory activity exerted by bilirubin on endothelial cells plays a role in preventing ischemia/reperfusion damage by reducing leukocyte infiltration, as discussed in section “Effects on Adhesion Molecule Expression: Implications for Obesity, Atherosclerosis and Chronic Inflammatory Diseases.”

Moreover, protection afforded by bilirubin against rejection of kidney transplantation has been demonstrated, and its antioxidant activity in preventing ischemia/reperfusion damage as well as its anti-inflammatory action that favors organ acceptance has been reviewed, highlighting the positive impact on renal hemodynamics (Sundararaghavan et al., 2018).

## Effects on Adhesion Molecule Expression: Implications for Obesity, Atherosclerosis and Chronic Inflammatory Diseases

The first evidence for the anti-inflammatory properties of bilirubin stems from Philip Hench who in 1938 described the remission of rheumatoid arthritis in the 94% of patients who developed jaundice (Hench, 1938). Even though Hench tried to correlate this phenomenon with an increased level of cortisol (Hench, 1949), this has never been demonstrated, and the anti-inflammatory effect of jaundice has been then proved to be due to UCB itself (Jangi et al., 2013). In this context, a negative correlation between bilirubin plasma concentration and clinical manifestations of other immune-mediated diseases such as MS (Peng et al., 2011) and SLE (Vítek et al., 2010) has been demonstrated. Moreover, an elevation of UCB between 2 and 12 mg/dl has been shown to reduce experimental autoimmune encephalomyelitis in an animal model of MS (Liu et al., 2008). However, it is important to note that UCB concentrations higher than 15–20 mg/dl always induce cytotoxicity.

The ability of UCB to modulate immunoglobulin production, native or acquired immunity as well as complement activity has been reviewed by others (Basiglio et al., 2009; Jangi et al., 2013), highlighting how UCB can impact neutrophil and macrophage phagocytosis, as well as antigen presenting functions to lymphocytes and the generation of pro-inflammatory cytokines. In this way, UCB exerts beneficial effects in different chronic inflammatory and autoimmune diseases, as discussed in section “Bilirubin and Chronic Inflammatory Diseases.” However, we here focus on the role played by endothelial cells, which are closely involved in the onset and development of inflammation.

### Bilirubin and Obesity

Endothelial dysfunction and inflammation characterize the development and progression of obesity, and mild hyperbilirubinemia contributes to a reduction of obesity especially during aging. These findings were particularly significant when abdominal obesity, triglyceridemia and hip and waist circumference were considered (Seyed Khoei et al., 2018). The same authors have shown that in Gilbert individuals expression level of p-AMPK, PPAR-α and γ, and PGC1-α in PBMCs were significantly higher in comparison to age- and gender-matched control subjects (Mölzer et al., 2016), whilst others have shown that UCB limits lipid deposition in adipose cells by binding PPAR-α nuclear receptors (Stec et al., 2016). The effect of UCB toward PPARα is discussed subsequently with a focus on atherosclerotic lesions (see section “Bilirubin and Atherosclerosis”).

Furthermore, a positive effect of CoPPIX-induced HMOX1 activation on reduction of body weight has been reported by Abraham et al. (2016). The same group, more recently focused on long-term endothelial HMOX1 activation in mice fed a HFD demonstrating the gene therapy to increase expression of HMOX1, reduced ICAM and VCAM expression, decreased serum markers of inflammation, such as IL-1 and TNFα, reduced the size of adipocytes and down-regulated PPARγ. Furthermore, these effects were prevented by using a specific inhibitor of HMOX1 activity, indirectly suggesting that the ability to restore a proper crosstalk between the vasculature and adipocytes depends on the metabolic products of endothelial HMOX1 activity (Peterson et al., 2019). Together these findings demonstrate that endothelial activation of HMOX1 could play an important role in the treatment/prevention of obesity, and further highlight the importance of AMPK-dependent signaling in mediating the anti-obesogenic effects of bilirubin.

### Bilirubin and Atherosclerosis

Endothelial activation and recruitment of inflammatory cells are two pivotal steps in the development of atherosclerotic lesions (Moore et al., 2013). The negative correlation between plasma concentrations of bilirubin and onset and progression of CVD is clearly demonstrated by numerous studies and is evident in individuals with GS (Hopkins et al., 1996; Vítek et al., 2002; Novotný and Vítek, 2003; Maruhashi et al., 2012; Vítek, 2017).

UCB has been shown to impair leukocyte migration by affecting endothelial adhesion molecules. The activation of endothelial cells induced by pro-inflammatory cytokines such as TNFα leads to the expression of adhesion molecules (Young et al., 2002). *In vitro* studies demonstrated that UCB prevents TNFα-induced leukocyte adhesion to endothelial cells by reducing the expression of E-selectin, VCAM and ICAM (Mazzone et al., 2009a) by impairing NF-kB nuclear translocation (Mazzone et al., 2009b). However, others maintain that bilirubin does not modulate expression of adhesion molecules but instead impairs adhesion molecule-dependent intracellular signals. Indeed, the signaling cascade activated by the binding of leukocyte integrins to VCAM and ICAM is known to generate ROS from the activity of NOX and XO, favoring leukocyte transmigration following activation of MMP2 (Wang and Doerschuk, 2000, 2002). Vogel et al. (2017) have reported that the intraperitoneal administration of bilirubin in LdL^–/–^ mice prevents atherosclerotic plaque formation by inhibiting leukocyte infiltration in vessel wall. These authors also confirmed *in vitro* that both exogenous bilirubin and HMOX1 derived bilirubin are highly efficient in reducing VCAM and ICAM dependent signals by quenching NOX4- and XO-derived ROS.

It has clearly been shown that UCB mimics the hypolipidemic activity of fenofibrate (Hinds and Stec, 2018). In addition, its ability to act as Selective PPAR Modulator (SPPARM) toward PPARα has been pointed out, confirming that, by enhancing lipid metabolism, bilirubin is able to reduce cholesterol deposition, decreasing plaque formation (Hinds and Stec, 2018). However, it has been also reported that UCB decreases the export of cholesterol from macrophages through the degradation of the ABCA1 (Wang et al., 2017). These data show that, in the vessel wall, the activity of bilirubin on lipid metabolism is complex and merits further investigation. However, serum UCB does exert a strong antioxidant activity at the level of atherosclerotic plaque by reducing the content of lipoperoxides in the lesions (Lapenna et al., 2018).

As recently reviewed, bilirubin prevent platelets aggregation due its ability to interfere with the surface expression of adhesion molecules and its antioxidant activity, thereby supporting a role played in the prevention of hypercoagulability and thrombosis (Kundur et al., 2015). Even though at high concentration (50–200 μM) UCB activates p38/MAPK favoring platelet apoptosis (NaveenKumar et al., 2015), the protective role of bilirubin against platelet aggregation has been confirmed in Gilbert individuals (Kundur et al., 2017), providing evidence for the inhibition of collagen-induced activation (Kundur et al., 2014).

### Bilirubin and Chronic Inflammatory Diseases

As mentioned before, the beneficial role of UCB in autoimmune and chronic inflammatory diseases is widely accepted. Indeed, there is clinical evidence that higher total serum bilirubin levels reduce the risk of rheumatoid arthritis (Fischman et al., 2010), Gilbert’s subjects are less likely to develop Crohn’s disease (de Vries et al., 2012), and patients with MS have low levels of total, conjugated and UCB (Peng et al., 2011, 2012), as highlighted in a previous extensive review (Jangi et al., 2013).

Focusing on the role played by endothelial cells in the context of chronic inflammation, it has been shown that bilirubin impairs the expression and the activity of adhesion molecules as documented in animal models of airways inflammation and inflammatory colitis. Indeed, intraperitoneal administration of bilirubin blocks the influx of leukocytes into the lungs of mice with OVA-induced asthma (Keshavan et al., 2005), and bilirubin is able to counteract iNOS expression and activity in colonic tissue by preventing leukocytes infiltration (Zucker et al., 2015). In both these studies, the molecular mechanism involves the ability of bilirubin to disrupt VCAM1-dependent signaling by quenching intracellular ROS.

## Effects on Angiogenesis: Implications for Wound Healing, Vascular Dysfunction in Pregnancy and Tumor Growth

Pro-angiogenetic activity of bilirubin has been demonstrated and related both to vascular activation of HMOX1 and to an increased plasma concentration of UCB. Favorable or detrimental actions can be associated with increased angiogenesis, depending on the different pathological conditions involved. Indeed, pro-angiogenetic activity can be desirable in repairing tissue damage or in preventing placental dysfunction (Chen and Zheng, 2014) but becomes deleterious when is linked to retinal degeneration in diabetes (Wang and Lo, 2018) or in the context of tumor growth (Viallard and Larrivée, 2017).

At a molecular level, pro-angiogenetic activity of bilirubin is achieved by the modulation of different pathways, some of which are not directly related to its antioxidant activity. *In vivo* and *in vitro* studies have shown that exogenous bilirubin promotes angiogenesis in response to ischemia via direct modulation of the PI3K/AKT pathway, which favors eNOS activation (Ikeda et al., 2015). The activation of AKT pathway in response to bilirubin exposure has been also been confirmed in other studies (Liu et al., 2015).

Furthermore, vascular HMOX1 induction favors the production of angiogenic factors, such as VEGF and IL-8, and decreases antiangiogenic factors such as VEGF soluble receptor (Dulak et al., 2008; Kim Y.-M. et al., 2011), with all HMOX1 metabolic products seeming to mediate these proangiogenic activities (Dulak et al., 2008; Kim Y.-M. et al., 2011). Moreover, the angiogenic potential of endothelial cells exposed to intermittent HG is restored by CoPPIX-induced HMOX1 activation to reduce ER stress (Maamoun et al., 2017). In this context, the protective role of HMOX1 in maintaining endothelial function by preventing both oxidative and ER stress has recently been reviewed (Maamoun et al., 2019). VEGF, in addition, is able to activate endothelial HMOX1 favoring angiogenesis, leading to positive feedback. However, the role played by HMOX1 in the vasculature is more complex, since it has been shown in VSMC that HMOX1-derived CO is able to increase VEGF production, whilst HMOX1-derived iron can inhibit VEGF, thus affecting endothelial responses in different ways (Dulak et al., 2002) which are not completely understood.

Furthermore, constitutive HMOX2 has been proposed to play an important role in angiogenesis. In HMOX2 null mice, an increase in corneal angiogenesis has been observed, which is paralleled by a down-regulation of HMOX1 (Seta et al., 2006). However, in another study HMOX2 deletion favors angiogenesis but, in this case, a parallel up-regulation of HMOX1 was observed (Bellner et al., 2009). Thus, the interplay between HMOX1 and HMOX2 seems to be more complex and merits further investigation.

### Bilirubin and Wound Healing

The process of wound healing is strictly dependent on tightly regulated angiogenesis. The role played by both HMOX1 (Lundvig et al., 2012) and HMOX2 (Halilovic et al., 2011) in wound healing has been well documented and related to the generation of CO and bilirubin. In particular, HMOX1 deficient mice exhibit less capability for wound healing compared to wild type mice due to a reduced ability to recruit endothelial progenitor cells (EPC) for capillary formation (Deshane et al., 2007). Interestingly, gene therapy performed using an hypoxia-regulated vector encoding for HMOX1 protects against oxidative damage and favors tissue regeneration by improving angiogenesis both *in vitro* and *in vivo*, avoiding the potential toxic effects due to iron overload (Jazwa et al., 2013). In this context, it has recently been reported that hyperbilirubinemic infants have an increased number of circulating endothelial progenitor cells (cEPC) which also exhibit an increased ability to proliferate, migrate and repair wounds. Indeed, tissues treated with conditioned medium derived from these cells up-regulate the expression of VEGF and IL-10 and reduce TNFα (Jabarpour et al., 2018). The involvement of bilirubin has also been documented in a model of cutaneous wounds, where i.p. administration of bilirubin (30 mg/kg) increases contraction and extracellular matrix deposition, with a down regulation of pro-inflammatory markers such as TNFα (Ahanger et al., 2016). Further evidence for a vasoactive role of bilirubin was reported by Ram and colleagues, who demonstrated that the topical treatment with 0.3% bilirubin ointment reduces oxidative stress in the granulation tissue of wounds in diabetic rats to favor wound healing (Ram et al., 2014, 2016). Other studies have underlined that, in chronic inflammation, HMOX1 could also be involved in the inhibition of leukocyte infiltration thereby favoring angiogenesis to complete tissue repair (Bussolati and Mason, 2006).

### Bilirubin and Vascular Dysfunctions in Pregnancy

The involvement of HMOX1 in placental development and pathology has been reviewed by Levytska et al. (2013) and also highlighted by others (George et al., 2011, 2013). HMOX1 deficiency is associated with the pathogenesis of pregnancy complications and PE (Zhao et al., 2008). In this context, studies with human umbilical vein endothelial cells and trophoblasts highlight the importance of bilirubin in angiogenesis, particularly in the regulation of spiral artery remodeling (Ha et al., 2015). Moreover, in choriocarcinoma cells, it has been shown that the up-regulation of HMOX1 or exposure to bilirubin prevents advanced glycation end-product induced generation of sFTL-1, the soluble decoy receptor for VEGF, known to play a critical role in the pathogenesis of PE (Jeong et al., 2014). However, it is worth noting that placenta-derived soluble factors involved in maternal hypertension act by increasing endothelin-1 production from endothelial cells. In this context, it has been shown that HMOX1 expression prevents endothelin-1 production in glomerular endothelial cells (Bakrania et al., 2018) and pharmacological activation of HMOX1 or treatment with bilirubin has been proposed to attenuate PE (Ramma and Ahmed, 2014).

### Bilirubin and Tumor Growth

In general, bilirubin is associated with anti-proliferative and anti-neoplastic activity. In fact, specific polymorphisms in UGT1A1 gene and the consequent hyperbilirubinemia are correlated positively with an increased overall survival in cancer patients (Vitek et al., 2019b), particularly considering colorectal (Jirásková et al., 2012), lung and breast cancers (Wagner et al., 2015). Moreover, reduced bilirubin levels are associated with increased cancer risk (Ching et al., 2002; Zucker et al., 2004). There are a few exceptions, for instance, a reported increase in the incidence of breast cancer in Gilbert individuals, which is related to specific polymorphisms of UGT1A1 promoter, as reviewed by Wagner et al. (2015).

At molecular level, an antigenotoxic activity of bilirubin has been hypothesized and a negative correlation between bilirubin concentration and DNA damage in epithelial cells has been documented (Wallner et al., 2012) even though, this has not been confirmed from an analysis of 8oxodGuo in PBMCs from GS individuals or Gunn rats (Wallner et al., 2013).

Thus, bilirubin not unlike other antioxidants, can counteract cancer onset and development by preventing DNA damage and the oxidative stress which plays a central role in carcinogenesis (Marengo et al., 2016).

Moreover, the anti-cancer proprieties of bilirubin have been documented, with bilirubin administration i.p. in mice increasing plasma bilirubin up to 40μM and drastically reducing colon cancer growth via activation of ERK1/2 (Ollinger et al., 2007). These findings have been confirmed by *in vitro* experiments showing that bilirubin concentrations >25μM exert pro-apoptotic activity whereas concentrations <25μM have no effect on cell viability or proliferation.

Nonetheless, it is important to note that once a tumor mass has developed, cancer cells can take advantage of antioxidants, especially when they derived from the activation of intrinsic adaptive pathways, and can be used for their growth and survival, favoring resistance to therapies and disease progression (Gorrini et al., 2013; Gill et al., 2016). This has been well documented for other antioxidants such as GSH (Traverso et al., 2013) and may apply to bilirubin. Indeed, role of HMOX1 in cancer progression has been highlighted and, even though some tissue specificity need to be considered, in many cancer types its expression correlates with tumor growth, aggressiveness, metastatic and angiogenetic potential, resistance to therapy, tumor escape, and poor prognosis (Furfaro et al., 2016; Nitti et al., 2017; Piras et al., 2017). It is important to note that HMOX1 and its metabolic byproducts can be involved in the generation of a permissive microenvironment, which is fundamental for cancer progression. In this context, a role for HMOX1 derived bilirubin has been implicated by Di Biase et al. (2016) and our group (Furfaro et al., 2019) in the progression of melanoma. Considering the role HMOX1 in reducing immune-surveillance, in favoring angiogenesis and invasiveness, it seems likely that some of these properties can be ascribed to the *in loco* generation of bilirubin.

Moreover, the involvement of HMOX1 in physiological angiogenesis in pregnancy and pathological angiogenesis in cancer has been proposed, since both share a necessity for a permissive microenvironment in which cell invasion, cytoprotection, angiogenesis and immune-escape are favored (Zhao et al., 2015). However, the specific role of bilirubin in this context has yet to be elucidated, providing a new field for investigation.

## Therapeutic Perspectives

Different therapeutic approaches aimed at elevating plasma bilirubin concentration as well as modulating cellular HMOX1 activity have been proposed for the treatment of many disorders, as widely reviewed by others (McCarty, 2007; Vitek et al., 2019b). Here, we focused on that ones in which the modulation of endothelial cell function is pivotal.

### Increasing the Level of Plasma Bilirubin

Elevating bilirubin plasma concentration needs to be considered carefully due to bilirubin’s well-known toxic effects. As discussed, bilirubin supplementation significantly decreases infarct size in animal models of ischemia damage (Ben-Amotz et al., 2014) as does BRT (Bakrania et al., 2016). Notably, in a mouse model of islet transplantation, bilirubin supplementation i.p. to a donor mouse as well as *in vitro* preconditioning of cells significantly suppresses the immune response and favors tolerance improving the outcomes (Adin et al., 2017). Furthermore, the use of nanoparticle of PEGylated bilirubin has been reported to protect liver tissue from ischemia-reperfusion damage (Kim et al., 2017) and to reduce lung inflammation (Kim et al., 2017). Notably, the use of bilirubin-coated stents reduces inflammation and endothelial activation preventing restenosis in porcine carotid arteries (Bae et al., 2018).

In humans, elevated plasma bilirubin levels, as side effects of drugs such as HIV PIs, have been proposed to improve endothelial function. For instance, 3-day atazanavir treatment in type II diabetes patients significantly ameliorates endothelial dysfunction (Dekker et al., 2011). Notably, three different HIV PIs, ritonavir, atazanavir and lopinavir, have been shown to be inducers of endothelial HMOX1. Moreover, HMOX1 induction is able to attenuate PI-dependent antiproliferative and inflammatory activity on endothelial cells primarily through the generation of bilirubin (Liu et al., 2016).

Importantly, therapeutic approaches leading to induce iatrogenic GS and to improve bioavailability of bilirubin might represent a valid strategy to prevent and ameliorate oxidative stress-related diseases, and McCarty (2007) already highlighted endothelial cells as crucially targeted by these therapies. More recently, the efficacy of different natural compounds which act by inhibiting UGT1A1 and elevate serum bilirubin has been confirmed, for instance silymarin, a seed extract of milk thistle (Silybum marianum), is able to protect against lipoperoxidation (Šuk et al., 2019) as are other natural compounds and herbal extracts (Vitek et al., 2019a). In addition, it is important to note that many natural compounds can increase bilirubin generation by activating HMOX1, as demonstrated that curcumin inducing HMOX1 expression and elevating bilirubin serum level, leading to protection against acute vascular inflammation both *in vivo* and *in vitro* (Xiao et al., 2018).

Furthermore, bilirubin analogs or related compounds can exert anti-proliferative effects as discussed earlier. In this context it is important to note that administration of natural tetrapyrrolic compounds structurally related to bilirubin, such as molecules extracted from *Spirulina platensis*, exert *in vitro* and *in vivo* actions including antiproliferative effects in cancer cells (Konícková et al., 2014). In addition, nanoparticles of both biotinylated and PEGylated bilirubin have recently been developed as drug delivery system improving the anti-angiogenetic efficacy of target tumor therapy (Lee et al., 2018; Yu et al., 2019).

### Modulation of Cytoprotective HMOX1

The role of HMOX1 activity in protection of endothelial cells against oxidative stress has been already highlighted (Kim Y.-M. et al., 2011) as has the opportunity to target HMOX1 to improve endothelial function and prevent CVD. However, induction of HMOX1 activity can be potentially harmful, mainly due to the generation of toxic amounts of iron. Thus, even though hemin is commonly used in experimental settings both *in vitro* and in animals to activate HMOX1, the use of the heme analogs such as heme arginate or hematin remains potentially toxic at the vascular level (Balla et al., 2000). However, other drugs such as niacin (Wu et al., 2012), statins (Grosser et al., 2004) and fenofibrates (Krönke et al., 2007) have been shown to be vasculoprotective via activation of HMOX1. Interestingly, valsartan induces HMOX1 expression in the aortic wall and increases serum bilirubin levels via down-regulation of AT1, leading to the reduction of intimal thickening (Li et al., 2014).

Finally, to achieve a more efficient up-regulation of HMOX1 and to avoid its pathological overexpression a molecular approach has been proposed: the use a hypoxia-regulated plasmid coding for HMOX1 to enhance vascular cell resistance *in vitro* and improve recovery from ischemia/reperfusion injury *in vivo* (Jazwa et al., 2013).

As far as tumor therapy is concerned, it has been reported that chlorophyll is able to inhibit HMOX1 expression and activity, exerting antiproliferative and antioxidative effects toward pancreatic cancer and inducing a significant reduction of pancreatic tumor size (Vanková et al., 2018). However, the efficacy of HMOX1 modulation specifically toward tumor angiogenesis has yet to be investigated.

## Conclusion and Future Research

**FIGURE 3:**
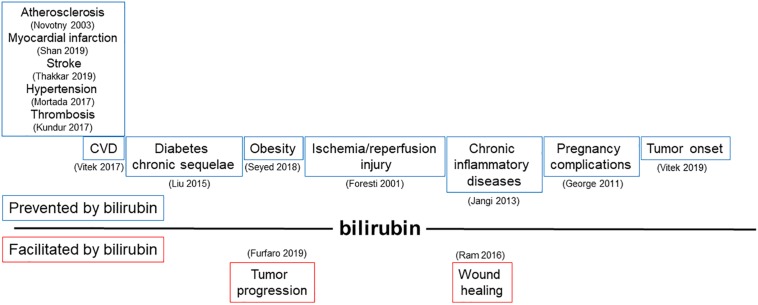
Modulation of bilirubin production can affect a broad range of pathologies driven by endothelial dysfunction. Schematic representation of pathophysiological processes influenced by the ability of bilirubin to modulate endothelial function. In the upper part, diseases prevented by bilirubin are shown; in the lower part, conditions favored by locally generated bilirubin are indicated. References in brackets.

Bilirubin, far from being just a waste product highly toxic for neurons, has been recognized as a powerful antioxidant and anti-inflammatory molecule. The potential application of bilirubin for the treatment of human diseases is driven by its ability to prevent endothelial dysfunction, thus affecting not only the treatment and prevention of CVDs (Ayer et al., 2016; Bulmer et al., 2018; Maruhashi et al., 2019), but also many other pathologies in which the alterations of endothelial cells has been proved to play a role (Figure 3 and Tables 1, 2).

**TABLE 1:** Bilirubin affects vascular cell functions: *In vivo* and *ex vivo* studies.

Experimental model	Plasma bilirubin concentration (mg/dl) or μM	Main findings	References
**Hypertension**			
C57BL/6J mice	0.9–1 mg/dl	Bilirubin i.v. infusion (37.2 mg/kg) or indinavir induce moderate hyperbilirubinemia and prevent AngII dependent hypertension	Vera et al., 2009
Gunn rats	117.9 ± 9.8 μM	Pathophysiological concentrations of UCB protects animals from hypertensive effects of AngII by reducing oxidative stress	Pflueger et al., 2005
Human plasma	15 μM	The addition of bilirubin to human plasma samples reduces peroxynitrite-induced protein modifications	Minetti et al., 1998
C57BL/6J mice	1–1.2 mg/dl	Moderate elevations in unconjugated bilirubin are able to preserve renal hemodynamics in AngII-dependent hypertension.	Vera and Stec, 2010
C57BL/6J mice	1–2 mg/dl	Mild hyperbilirubinemia induced by UGTA1A1 antisense decreases AngII-induced hypertension inhibition superoxide generation	Stec et al., 2013
Old male SHRs or WKY rats	Serum content not modified	Up-regulation of HMOX1, through the generation of bilirubin, improves endothelial function favors vessel relaxation in a rat model of spontaneous hypertension	Li et al., 2013
**Diabetes**			
Diabetic patients with or without GS	1.3–1.6 mg/dl	Patients with diabetes and GS show a lower prevalence of vascular complications than diabetic patients	Inoguchi et al., 2007
Diabetic patients (Hisayama study)	0.89–1.1 mg/dl	Patients with plasma bilirubin concentration in the highest quartile show a reduced prevalence of diabetic retinopathy	Yasuda et al., 2011
Diabetic patients (type 2 DM)	0.80 ± 0.39 mg/dl	Serum total bilirubin concentration negatively correlated with the severity of retinopathy	Sekioka et al., 2015
STZ-induced diabetic rats	3.3 mg/dl	Up-regulation of HMOX1 activity induced by CoPPIX by increasing bilirubin concentration reduces endothelial damage in diabetic animals	Quan et al., 2004
STZ-induced diabetic Gunn rats	7.01 ± 0.43 mg/dl	Hyperbilirubinemic rats are protects against renal diabetic complications	Fujii et al., 2010
db/db mice	Not significant increases	Biliverdin (5 mg/kg) protects diabetic mice toward renal complications	Fujii et al., 2010
db/db mice	200 nM	HMOX1 induction and BLVRA activation prevent endothelial damage in diabetes, through the modulation of Akt pathway	Liu et al., 2015
db/db and DIO mice	Not evaluated	HMOX1 activation (CoPPIX 10–20 mg/kg, i.p.) and Bilirubin (20 μmoli/kg i.p.) reduce hyperglycemia and increase insulin sensitivity	Dong et al., 2014
T2DM patients	64 ± 21 μM	Short-term treatment with atazanavir inhibits UGT1A1 activity induces a mild hyperbilirubinemia and ameliorates endothelial functions	Dekker et al., 2011
**Ischemia/reperfusion damage**			
Healthy human subjects	<0.5 mg/dl	Low levels of plasma bilirubin are associated with a high prevalence of ischemic modifications in brain white matter	Higuchi et al., 2018
MCAO rats	Not evaluated	Biliverdin administration (35 mg/kg i.p.) significantly reduces the cerebral infarct size	Zou et al., 2019
“*Ex vivo*” perfused rat hearts	Not applicable	Treatment with bilirubin ditaurate (BRT, 50 μM) prevents ischemia/reperfusion damage. The infarct size shows negative correlation with BRT tissue content	Bakrania et al., 2016
Rat model of coronary ischemia	Not evaluated	Intraperitoneal injection of bilirubin (10 mg/kg) decreases heart infarct size	Ben-Amotz et al., 2014
C57BL/6 mice	Not evaluated	Preconditioning performed using nanoparticles of PEG-bilirubin (10 mg/kg) attenuates IRI-associated hepatocellular injury	Kim et al., 2017
**Obesity**			
GS and age-/gender-matched healthy controls and obese female type 2 diabetes patients	>17.1 μM (1 mg/dl)	Mild hyperbilirubinemia protects from age-related weight gain and dyslipidaemia.	Seyed Khoei et al., 2018
GS subjects	35.3 ± 1.04 μM (male) 28.9 ± 6.8 μM (female)	GS subjects show higher expression level of p-AMPK, PPARα and γ and PGC1α in PMBCs in comparison to age- and gender –matched control subjects. They are less likely to contract metabolic diseases or die prematurely	Mölzer et al., 2016
PPARα knockout		Bilirubin (30 mg/kg) directly binds to activate PPARα and limits lipid deposition in adipose cells	Stec et al., 2016
C57 male mice		HMOX1 induction by CoPPIX (5 mg/100 gr subcutaneous) or HMOX1 endothelial overexpression by gene therapy in HFD mice reduce inflammation markers, down-regulate PPARγ and reduce adipocytes size	Peterson et al., 2019
**Atherosclerosis**			
GS subjects	29.2 ± 11.6 μM	Mild hyperbilirubinemia contribute to reduce oxidative stress and vascular complications in atherosclerotic patients with GS	Maruhashi et al., 2012
GS subjects	32.6 ±13.5 μM	Mild hyperbilirubinemia protects GS subjects from ischemic heart disease	Vítek et al., 2002
Male atherosclerotic patients	//	Meta-analysis of 11 published studies proves the inverse relationship between serum bilirubin levels and atherosclerosis	Novotný and Vítek, 2003
Early familial CAD patients	8.9 ± 6.1 μM(CAD) 12.4 ± 8.1 μM(ctr)	CAD risk is negatively correlated with bilirubin concentration	Hopkins et al., 1996
Ldlr^–/–^ mice	1.7 mg/dl	Intraperitoneal injection of bilirubin reduces VCAM and ICAM signaling in endothelial cells preventing plaque formation	Vogel et al., 2017
Patients with carotid atherosclerotic plaques	13.6 ± 3 μM	Serum concentration of bilirubin (total, direct and indirect) is correlated with the reduction of lipoperoxides in the lesions	Lapenna et al., 2018
GS subjects	23 ± 5 μM	Increased levels of circulating bilirubin inhibits platelet aggregation and granule release	Kundur et al., 2014
**Chronic inflammation**			
MS patients	<13.97 μM	Patients with long duration MS show low levels of bilirubin plasma concentration	Peng et al., 2011
SLE patients	7.1 ± 5.3 μM	Low serum bilirubin represented a strong predictor of the manifestation of SLE symptoms Subjects with higher serum bilirubin levels, such as those with Gilbert’s syndrome, might be protected from the development of SLE.	Vítek et al., 2010
RA patients	>11 μM	Higher serum total bilirubin level is negatively associated with RA	Fischman et al., 2010
Crohn’s patients	>23 μM	GS confers a protective effect on the development of Crohn’s disease	de Vries et al., 2012
SJL/J mice and Lewis rats	Not evaluated	Bilirubin treatment (2 × 100 mg/kg per day i.p.) successfully prevented the development of chronic EAE. Treatment with ZnPP exacerbates acute EAE	Liu et al., 2008
C57BL6/J mice	Not evaluated	Bilirubin (30 mg/kg i.p.) suppresses colonic inflammation, induced by the oral administration of DSS, by preventing leukocytes infiltration	Zucker et al., 2015
C57BL/6J	Not evaluated	Bilirubin treatment (30 mg/kg i.p.) blocks the influx of leukocytes into the lungs of mice with OVA-induced asthma	Keshavan et al., 2005
**Wound healing**			
C57BL/6J	0.55 ±0.04 mg/dl (after 1 h i.p.)	Bilirubin treatment (5 mg/kg i.p.) enhance blood flow recovery in response to ischemia by promoting angiogenesis through endothelial cells activation via Akt-eNOS-dependent manner	Ikeda et al., 2015
HMOX1^–/–^ mice	Not evaluated	HMOX1null mice show less capability of wound healing compared to WT mice due to a reduced endothelial migration and neovascularization	Deshane et al., 2007
HMOX1^–/–^ mice	Not evaluated	Gene therapy by using pHRE-HO-1 vector protects against oxidative stress, improves angiogenesis and favors tissue regeneration	Jazwa et al., 2013
Wistar rats	Not evaluated	Administration of Bilirubin (i.p. 30 mg/kg) favors cutaneous wound healing by a pro-healing modulation of pro-inflammatory/anti-inflammatory cytokines, adhesion molecule and favoring angiogenesis	Ahanger et al., 2016
STZ Wistar rats	Not applicable	Bilirubin treatment (0.3% ointment) accelerates the timely progression of wound healing by modulating expression of cytokines and growth factors (HIF-1α, VEGF, TGF-β1, SDF-1α, TNFα, IL-1β, IL-10 and MMP-9) promotes angiogenesis, reduces inflammation and improves maturation of wound	Ram et al., 2016
STZ Wistar rats	Not applicable	Bilirubin treatment (0.3% ointment) markedly improved the antioxidant profile of the diabetic wound and accelerates cutaneous wound healing	Ram et al., 2014
**Pregnancy complications**			
FVB pregnant mice	Not evaluated	Pharmacological inhibition of HMOX1 (induced by SnPP 30 μmoli/kg i.v.) is associated with pathogenesis of pregnancy complications and preeclampsia	Zhao et al., 2008
**Tumor**			
Cancer patients	>17 μM	UGT1A1 gene polymorfism is positively correlate with overall survival in cancer patients	Vitek et al., 2019b
GI and CRC patients	0.52 ±0.017 mg/dl	Increase in serum bilirubin level is associated with a markedly decreased prevalence of CRC	Zucker et al., 2004
CRC patients	9.8 μM	Patients with CRC have lower serum bilirubin levels: 1 μmol/L decrease in serum bilirubin is associated with a 7% increase in CRC risk. UGT1A1^∗^28 allele carrier status is associated with a 20% decrease in risk of CRC	Jirásková et al., 2012
GS subjects	32 ± 13.6 μM	Older subjects with GS show decreased DNA damage in epithelial tissue. No correlation have been found with DNA damage in lymphocytes	Wallner et al., 2012
BALB/c nude mice	35–40 μM	Treatment with bilirubin (25 mg/kg i.p.) reduces colon cancer growth via activation of ERK1/2	Ollinger et al., 2007
BALB/c-nude mice	Not evaluated	Fasting-mimicking diet (FMD) or short-term starvation (STS) reduces HMOX1 expression in cancer and sensitizes cancer cells to chemotherapy	Di Biase et al., 2016
**Aging**			
12-month-old Gunn rats	75 ±18 μM	Mildly elevated levels of bilirubin improve anthropometric and metabolic parameters in hyperbilirubinemic old rats respect to normobilirubinemic	Zelenka et al., 2016
GS subjects	33.12 ±9.86 μM	GS subjects have on average longer telomeres compared to age- and gender-matched control	Tosevska et al., 2016
C57BL/6 mice	Not evaluated	Hemin-induced HMOX1 upregulation, through the generation of both CO and bilirubin, limits cardiomyocytes senescence	Shan et al., 2019

**In vivo* and *ex vivo* studies. AngII, angiotensin II; BRT, bilirubin ditaurate; BLVRA, biliverdin reductase; CAD, coronary artery disease; CO, carbon monoxide; CoPPIX, cobalt protoporphyrin IX; CRC, colorectal cancer patients; db/db, diabetic mice; DIO, diet-induced obese; DM, diabetes mellitus; DSS, dextran sodium sulfate; EAE, experimental autoimmune encephalomyelitis; FMD, fasting-mimicking diet; FVB, friend leukemia virus B; GI, gastro-intestinal; GS, Gilbert Syndrome; HFD, high fat diet; IRI, ischemia/reperfusion injury; Ldlr^–/–^, low-density lipoprotein receptor-deficient mice; MCAO, middle cerebral artery occlusion; MS, multiple sclerosis; OVA, ovalbumin; PMBCs, peripheral blood mononuclear cells; RA, rheumatoid arthritis; SHR, spontaneously hypertensive rat; SJL, Swiss Jim Lambert mice; SLE, systemic lupus erythematosus; SnPP, tin protoporphyrin IX; STS, short-term starvation; STZ, streptozocin; UCB, unconjugated bilirubin; UTG1A1, uridine diphpspho-glucuronosyl transferase 1A1; ZnPP, zinc protoporphyrin IX; WKI, Wistar Kyoto rat.*

**TABLE 2:** Bilirubin affects vascular cell functions: *In vitro* studies.

Experimental model	Bilirubin concentration (mg/dl) or μM in cell culture media	Main findings	References
**Human EC**			
HUVECs	10–20 μM bilirubin	Bilirubin inhibits migration of THP-1 monocytes across activated HUVEC monolayers	Vogel et al., 2017
HUVECs	20 μM	Bilirubin markedly inhibited the migration of Jurkat cells across TNFα-stimulated HUVEC monolayers by impairing VCAM1 signaling	Zucker et al., 2015
HUVECs	Not evaluated	HMOX1 induction (10 μM CoPPIX) prevents high glucose-induced reduction in NO release and enhanced VEGF-A expression	Maamoun et al., 2017
HUVECs	50 μM	Bilirubin promoted proliferation of endothelial cells and also affects invasion capability of cells from trophoblast	Ha et al., 2015
HUVECs and VSMCs	Not evaluated	PPAR ligands induce HMOX1 and block the inflammatory response in vascular cells	Krönke et al., 2007
HUVECs, HAECs, HDMECs	10 μM	HIV protease inhibitors (PIs) induce HMOX1 that, by generating bilirubin, counteract the anti-proliferative and inflammatory actions of PIs	Liu et al., 2016
ECV304	Not evaluated	Statins increase HMOX1 expression	Grosser et al., 2004
HMEC-1	Not evaluated	Cell transfection with plasmid vector (pHRE-HO-1) carrying human HMOX1 driven by three hypoxia response elements (HREs) and cultured in 0.5% oxygen, by up-regulating HMOX1, effectively protects against oxidative stress and promotes angiogenesis	Jazwa et al., 2013
HBMECs	50 or 100 μM UCB in presence of HSA	Short-term exposure to UCB activates endothelial cells and late-term exposure to UCB increases paracellular permeability, overall increasing endothelial damage	Palmela et al., 2012
HAECs	1–10 μM	Bilirubin increases AKT dependent eNOS phosphorylation and favors leukocyte adhesion migration and tube formation	Ikeda et al., 2015
HCAECs	1, 5, and 10 μM bilirubin	Niacin increases HMOX1 expression and inhibits TNFα induced endothelial inflammation	Wu et al., 2012
HGEnCs	10 μM	HMOX1 induction (25 μM CoPPIX) as well as bilirubin supplementation directly reduce ET-1 generation	Bakrania et al., 2018
cEPCs	10–20 mg/dl	Progenitor endothelial cells induced to proliferate when exposed to 5 mg/dl bilirubin; higher concentrations (up to 20 mg/dl) induce cell death	Jabarpour et al., 2018
EA.hy926	0.5–100 μM	Exogenous bilirubin increases endothelial antioxidant activity as well as HMOX1-dependent bilirubin generation	Ziberna et al., 2016
Commercially EC	Not evaluated	EC-transfected with HMOX1 release substances that increase healthy adipocytes	Peterson et al., 2019
**Human and non-human EC**			
HUVECs and H5V cells	0.015 μM < Bf < 0.030 μM	UCB, at clinically relevant concentrations, limits over-expression of adhesion molecules and inhibits PMN-endothelial adhesion induced by pro-inflammatory cytokine TNFα, even though UCB itself does not alter expression of these adhesion molecules. Inhibition NF-kappaB transduction pathway	Mazzone et al., 2009a, b
HAECs and mAECs	Not evaluated	In HAECs and in primary mAECs from HMOX1^+/+^ and HMOX1^–/–^, SDF-1 (100-200 ng/ml) favors angiogenesis through the induction of HMOX1	Deshane et al., 2007
**Non-human EC**			
mHEVa and mHEVc	10–20 μM	Bilirubin inhibits leukocyte transmigration across endothelial cell monolayer	Keshavan et al., 2005
CMVECs	1 μM	Bilirubin supplementation prevent endothelial apoptosis induced by TNFα by reducing NOX4-derived ROS	Basuroy et al., 2009
bEnd.3 and MS1	1–40 μM	Endothelial cells derived from BBB are more sensitive to UCB pro-apoptotic effect than endothelial cells from pancreas	Kapitulnik et al., 2012
mAECs	Not evaluated	Primary endothelial cells from HMOX2^–/–^ mice show an increased oxidative stress, inflammation and excessive angiogenesis	Bellner et al., 2009
BAEC	1 μM	Bilirubin supplementation restores cell protection against acute high glucose treatment in endothelial cells exposed to HMOX1 inhibitor, preventing HNE production	He et al., 2015
**Other cells from cardiovascular system**			
Bovine vascular smooth-muscle cells	0.5–5 μM	Short term treatment with bilirubin as well as HMOX1 induction (25–200 μM hemin) protect against oxidant-mediated damage	Clark et al., 2000
H9c2	0.5 μM	Bilirubin treatment as well asHMOX1 induction (5 μM hemin) protects against hypoxia/reoxygenation	Foresti et al., 2001
Primary mice cardiomyocytes	20 μM	HMOX1 induction (10 μM hemin) as well as bilirubin supplementation limits senescence	Shan et al., 2019

*BAEC, bovine aortic endothelial cells; b.End3, murine brain microvascular endothelial cell line; Bf, free bilirubin; BBB, blood-brain barrier; cEPCs, circulating endothelial progenitor cells; CMVECs, cerebral microvascular endothelial cells; COPPIX, cobalt protoporphyryn IX; EA.hy926, human endothelial cell line; EC, endothelial cells; ECV304, human endothelial cells derived from umbilical cord; ET-1, vascular endothelin-1; HAECs, human aortic endothelial cells; HBMECs, human brain microvascular endothelial cells; HCAECs, human coronary artery endothelial cells; HDMECs, human dermal microvascular endothelial cells; HGEnCs, immortalized human glomerular endothelial cells; HMEC-1, human microvascular endothelial cells; HREs, hypoxia response elements; HUVECs, human umbilical vein endothelial cells; H5V, murine heart endothelial immortalized cells; H9c2, rat cardiomyocytes; mAECs, mouse aortic endothelial cells; mHEVa/c, murine high endothelial venule-like cells; MS1, microvascular endothelial cell line; PIs, protease inhibitors; PMN, polymorphonuclear neutrophils; ROS, reactive oxygen species; SDF-1, stromal cell-derived factor 1; UCB, unconjugated bilirubin; VSMC, vascular smooth muscle cells.*

As both increasing plasma levels of bilirubin and up-regulating HMOX1 could lead to toxic effects, the discovery of new strategies to reduce its toxic potential to maintain therapeutic effectiveness will be key for the treatment/prevention of endothelial dysfunction. Finally, it is worth noting that senescence and aging processes, strongly associated with accumulation of oxidative damage, are reduced/slowed by bilirubin. Notably, has been recently highlighted the negative relationship among bilirubin concentration and cancer mortality, even though the relationship is missing among bilirubin and CVD mortality (Vitek et al., 2019b). It is conceivable that larger studies focused on older population are needed to explain this lack of association. Indeed, the inverse correlation between plasma UCB levels and the prevalence of obesity has be proved to be stronger in older subjects (Seyed Khoei et al., 2018).

Nonetheless, it has been proved that in Gilbert subjects and in Gunn rats both mitochondrial and cytosolic ROS production is reduced leading to a decrease of chronic inflammation markers and better anthropometric parameters (Zelenka et al., 2016). Furthermore, telomere shortening is slower in patients with mild hyperbilirubinemia, implicating a correlation with reduced oxidative stress and blood markers of inflammation (Tosevska et al., 2016). Furthermore, in senescent fibroblasts the activity of BLVRA is significantly reduced in comparison to cells derived from younger donors, suggesting that a deficit of bilirubin may be responsible for increased oxidative stress and DNA damage (Kim S. Y. et al., 2011). Moreover, hemin-induced HMOX1 up-regulation limits cardiomyocyte senescence both *in vitro* and in aged mice via the generation of both CO and bilirubin (Shan et al., 2019). Thus, the careful modulation of HMOX1 activity and bilirubin generation offers a promising target in the context of prevention and treatment of endothelial dysfunction but also with an aim to ensure healthy aging.

## Author Contributions

All authors conceived and wrote the manuscript, and revised the literature. MN and GM provided financial support.

## Conflict of Interest

The authors declare that the research was conducted in the absence of any commercial or financial relationships that could be construed as a potential conflict of interest.

## Abbreviations

8-oxodGuo: 8-oxo-7,8-dihydro-2’-deoxyguanosine
ALA: 5-aminolevulinic acid
ABCA1: ATP-binding cassette transporter A
AngII: angiotensin II
AT1: angiotensin type 1 receptor
BBB: blood-brain barrier
BRT: bilirubin ditaurate
BLVRA: biliverdin reductase
CO: carbon monoxide
CoPPIX: cobalt protoporphyrin IX
CVD: cardiovascular disease
DM: diabetes mellitus
EDH: endothelial dependent hyperpolarization
EPC/cEPC: endothelial progenitor cells/circulating endothelial progenitor cells
FABPs: fatty acid binding proteins
GS: Gilbert syndrome
HFD: high fat diet
HG: high glucose
HMOX1: heme oxygenase 1
HMOX2: heme oxygenase 2
HNE: hydroxynonenal
MCAO: middle cerebral artery occlusion
MMP2: metalloproteinases
MS: multiple sclerosis
NOXs: NADPH oxidases
NSCLC: non-small cell lung cancer
OATP: organic anion transport binding protein
OVA: ovalbumin
PE: preeclampsia
PI: protease inhibitor
PMBCs: peripheral blood mononuclear cells
ROS: reactive oxygen species
SLE: systemic lupus erythematosus
UCB: unconjugated bilirubin
UTG1A1: uridine diphpspho-glucuronosyl transferase 1A1
VSMC: vascular smooth muscle cells
XO: xanthine oxidase

## References

[B1] Abraham N. G. Junge J. M. Drummond G. S. (2016). Translational significance of heme oxygenase in obesity and metabolic syndrome. *Trends Pharmacol. Sci.* 37 17–36. 10.1016/j.tips.2015.09.003 26515032 PMC5488647

[B2] Abu-Bakar A. Arthur D. M. Wikman A. S. Rahnasto M. Juvonen R. O. Vepsäläinen J. (2012). Metabolism of bilirubin by human cytochrome P450 2A6. *Toxicol. Appl. Pharmacol.* 261 50–58. 10.1016/j.taap.2012.03.010 22465937

[B3] Adin C. A. VanGundy Z. C. Papenfuss T. L. Xu F. Ghanem M. Lakey J. (2017). Physiologic doses of bilirubin contribute to tolerance of islet transplants by suppressing the innate immune response. *Cell Transplant.* 26 11–21. 10.3727/096368916X692096 27393133 PMC5657680

[B4] Ahanger A. A. Leo M. D. Gopal A. Kant V. Tandan S. K. Kumar D. (2016). Pro-healing effects of bilirubin in open excision wound model in rats. *Int. Wound J.* 13 398–402. 10.1111/iwj.12319 24947136 PMC7950029

[B5] Ayer A. Zarjou A. Agarwal A. Stocker R. (2016). Heme oxygenases in cardiovascular health and disease. *Physiol. Rev.* 96 1449–1508. 10.1152/physrev.00003.2016 27604527 PMC5504454

[B6] Bae I.-H. Park D. S. Lee S.-Y. Jang E.-J. Shim J.-W. Lim K.-S. (2018). Bilirubin coating attenuates the inflammatory response to everolimus-coated stents. *J. Biomed Mater. Res. B Appl. Biomater.* 106 1486–1495. 10.1002/jbm.b.33955 28691192

[B7] Bakrania B. Du Toit E. F. Wagner K.-H. Headrick J. P. Bulmer A. C. (2016). Pre- or post-ischemic bilirubin ditaurate treatment reduces oxidative tissue damage and improves cardiac function. *Int. J. Cardiol.* 202 27–33. 10.1016/j.ijcard.2015.08.192 26386915

[B8] Bakrania B. A. Spradley F. T. Satchell S. C. Stec D. E. Rimoldi J. M. Gadepalli R. S. V. (2018). Heme oxygenase-1 is a potent inhibitor of placental ischemia-mediated endothelin-1 production in cultured human glomerular endothelial cells. *Am. J. Physiol. Regul. Integr. Comp. Physiol.* 314 R427–R432. 10.1152/ajpregu.00370.2017 29212810 PMC5899255

[B9] Balla J. Balla G. Jeney V. Kakuk G. Jacob H. S. Vercellotti G. M. (2000). Ferriporphyrins and endothelium: a 2-edged sword-promotion of oxidation and induction of cytoprotectants. *Blood* 95 3442–3450. 10.1182/blood.v95.11.3442.011k51_3442_3450 10828027

[B10] Balla J. Vercellotti G. M. Jeney V. Yachie A. Varga Z. Jacob H. S. (2007). Heme, heme oxygenase, and ferritin: how the vascular endothelium survives (and dies) in an iron-rich environment. *Antioxid. Redox Signal.* 9 2119–2137. 10.1089/ars.2007.1787 17767398

[B11] Baranano D. E. Rao M. Ferris C. D. Snyder S. H. (2002). Biliverdin reductase: a major physiologic cytoprotectant. *Proc. Natl. Acad. Sci. U.S.A* 99 16093–16098. 10.1073/pnas.252626999 12456881 PMC138570

[B12] Basiglio C. L. Arriaga S. M. Pelusa F. Almará A. M. Kapitulnik J. Mottino A. D. (2009). Complement activation and disease: protective effects of hyperbilirubinaemia. *Clin. Sci. Lond. Engl.* 1979 99–113. 10.1042/CS20080540 19807696

[B13] Basuroy S. Bhattacharya S. Leffler C. W. Parfenova H. (2009). Nox4 NADPH oxidase mediates oxidative stress and apoptosis caused by TNF-alpha in cerebral vascular endothelial cells. *Am. J. Physiol Cell Physiol.* 296 C422–C432. 10.1152/ajpcell.00381.2008 19118162 PMC2660262

[B14] Bellarosa C. Bortolussi G. Tiribelli C. (2009). The role of ABC transporters in protecting cells from bilirubin toxicity. *Curr. Pharm. Des.* 15 2884–2892. 10.2174/138161209789058246 19754365

[B15] Bellner L. Martinelli L. Halilovic A. Patil K. Puri N. Dunn M. W. (2009). Heme oxygenase-2 deletion causes endothelial cell activation marked by oxidative stress, inflammation, and angiogenesis. *J. Pharmacol. Exp. Ther.* 331 925–932. 10.1124/jpet.109.158352 19773531 PMC2784722

[B16] Ben-Amotz R. Bonagura J. Velayutham M. Hamlin R. Burns P. Adin C. (2014). Intraperitoneal bilirubin administration decreases infarct area in a rat coronary ischemia/reperfusion model. *Front. Physiol.* 5:53. 10.3389/fphys.2014.00053 24600401 PMC3927123

[B17] Brito M. A. Palmela I. Cardoso F. L. Sá-Pereira I. Brites D. (2014). Blood-brain barrier and bilirubin: clinical aspects and experimental data. *Arch. Med. Res.* 45 660–676. 10.1016/j.arcmed.2014.11.015 25475697

[B18] Bulmer A. C. Bakrania B. Du Toit E. F. Boon A.-C. Clark P. J. Powell L. W. (2018). Bilirubin acts as a multipotent guardian of cardiovascular integrity: more than just a radical idea. *Am. J. Physiol. Heart Circ. Physiol.* 315 H429–H447. 10.1152/ajpheart.00417.2017 29600900

[B19] Bussolati B. Mason J. C. (2006). Dual role of VEGF-induced heme-oxygenase-1 in angiogenesis. *Antioxid. Redox Signal.* 8 1153–1163. 10.1089/ars.2006.8.1153 16910763

[B20] Calay D. Mason J. C. (2014). The multifunctional role and therapeutic potential of HO-1 in the vascular endothelium. *Antioxid. Redox Signal.* 20 1789–1809. 10.1089/ars.2013.5659 24131232

[B21] Chen D.-B. Zheng J. (2014). Regulation of placental angiogenesis. *Microcirc. N. Y. N.* 21 15–25. 10.1111/micc.12093 23981199 PMC5589442

[B22] Chen R.-J. Yuan H.-H. Zhang T.-Y. Wang Z.-Z. Hu A.-K. Wu L.-L. (2014). Heme oxygenase-2 suppress TNF-α and IL6 expression via TLR4/MyD88-dependent signaling pathway in mouse cerebral vascular endothelial cells. *Mol. Neurobiol.* 50 971–978. 10.1007/s12035-014-8693-x 24788682

[B23] Ching S. Ingram D. Hahnel R. Beilby J. Rossi E. (2002). Serum levels of micronutrients, antioxidants and total antioxidant status predict risk of breast cancer in a case control study. *J. Nutr.* 132 303–306. 10.1093/jn/132.2.303 11823595

[B24] Clark J. E. Foresti R. Green C. J. Motterlini R. (2000). Dynamics of haem oxygenase-1 expression and bilirubin production in cellular protection against oxidative stress. *Biochem. J.* 348(Pt 3), 615–619. 10.1042/bj3480615 10839994 PMC1221105

[B25] Datla S. R. Dusting G. J. Mori T. A. Taylor C. J. Croft K. D. Jiang F. (2007). Induction of heme oxygenase-1 in vivo suppresses NADPH oxidase derived oxidative stress. *Hypertens Dallas Tex* 1979 636–642. 10.1161/HYPERTENSIONAHA.107.092296 17679649

[B26] de Vries H. S. Te Morsche R. H. M. Jenniskens K. Peters W. H. M. de Jong D. J. (2012). A functional polymorphism in UGT1A1 related to hyperbilirubinemia is associated with a decreased risk for Crohn’s disease. *J. Crohns Colitis* 6 597–602. 10.1016/j.crohns.2011.11.010 22398043

[B27] Dekker D. Dorresteijn M. J. Pijnenburg M. Heemskerk S. Rasing-Hoogveld A. Burger D. M. (2011). The bilirubin-increasing drug atazanavir improves endothelial function in patients with type 2 diabetes mellitus. *Arter. Thromb. Vasc. Biol.* 31 458–463. 10.1161/ATVBAHA.110.211789 21088253

[B28] Deshane J. Chen S. Caballero S. Grochot-Przeczek A. Was H. Li Calzi S. (2007). Stromal cell-derived factor 1 promotes angiogenesis via a heme oxygenase 1-dependent mechanism. *J. Exp. Med.* 204 605–618. 10.1084/jem.20061609 17339405 PMC1855437

[B29] Di Biase S. Lee C. Brandhorst S. Manes B. Buono R. Cheng C. W. (2016). Fasting-mimicking diet reduces HO-1 to promote T cell-mediated tumor cytotoxicity. *Cancer Cell* 30 136–146. 10.1016/j.ccell.2016.06.005 27411588 PMC5388544

[B30] Dong H. Huang H. Yun X. Kim D. Yue Y. Wu H. (2014). Bilirubin increases insulin sensitivity in leptin-receptor deficient and diet-induced obese mice through suppression of ER stress and chronic inflammation. *Endocrinology* 155 818–828. 10.1210/en.2013-1667 24424052 PMC3929745

[B31] Doré S. Takahashi M. Ferris C. D. Zakhary R. Hester L. D. Guastella D. (1999). Bilirubin, formed by activation of heme oxygenase-2, protects neurons against oxidative stress injury. *Proc. Natl. Acad. Sci. U.S.A.* 96 2445–2450. 10.1073/pnas.96.5.2445 10051662 PMC26804

[B32] Dulak J. Deshane J. Jozkowicz A. Agarwal A. (2008). Heme oxygenase-1 and carbon monoxide in vascular pathobiology: focus on angiogenesis. *Circulation* 117 231–241. 10.1161/CIRCULATIONAHA.107.698316 18195184 PMC5536198

[B33] Dulak J. Józkowicz A. Foresti R. Kasza A. Frick M. Huk I. (2002). Heme oxygenase activity modulates vascular endothelial growth factor synthesis in vascular smooth muscle cells. *Antioxid. Redox Signal.* 4 229–240. 10.1089/152308602753666280 12006174

[B34] Engerman R. L. Meyer R. K. (1959). Anti-inflammatory effect of obstructive jaundice in rats. *Ann. Rheum. Dis.* 18 309–312. 10.1136/ard.18.4.309 13820359 PMC1007122

[B35] Erdogan D. Gullu H. Yildirim E. Tok D. Kirbas I. Ciftci O. (2006). Low serum bilirubin levels are independently and inversely related to impaired flow-mediated vasodilation and increased carotid intima-media thickness in both men and women. *Atherosclerosis* 184 431–437. 10.1016/j.atherosclerosis.2005.05.011 15979081

[B36] Fischman D. Valluri A. Gorrepati V. S. Murphy M. E. Peters I. Cheriyath P. (2010). Bilirubin as a protective factor for rheumatoid arthritis: an NHANES study of 2003 - 2006 data. *J. Clin. Med. Res.* 2 256–260. 10.4021/jocmr444w 22043258 PMC3194029

[B37] Foresti R. Goatly H. Green C. J. Motterlini R. (2001). Role of heme oxygenase-1 in hypoxia-reoxygenation: requirement of substrate heme to promote cardioprotection. *Am. J. Physiol. Heart Circ. Physiol.* 281 H1976–H1984. 11668058 10.1152/ajpheart.2001.281.5.H1976

[B38] Foresti R. Hoque M. Bains S. Green C. J. Motterlini R. (2003). Haem and nitric oxide: synergism in the modulation of the endothelial haem oxygenase-1 pathway. *Biochem. J.* 372 381–390. 10.1042/BJ20021516 12622689 PMC1223420

[B39] Foresti R. Sarathchandra P. Clark J. E. Green C. J. Motterlini R. (1999). Peroxynitrite induces haem oxygenase-1 in vascular endothelial cells: a link to apoptosis. *Biochem. J.* 339(Pt 3), 729–736. 10.1042/bj3390729 10215613 PMC1220210

[B40] Fujii M. Inoguchi T. Sasaki S. Maeda Y. Zheng J. Kobayashi K. (2010). Bilirubin and biliverdin protect rodents against diabetic nephropathy by downregulating NAD(P)H oxidase. *Kidney Int.* 78 905–919. 10.1038/ki.2010.265 20686447

[B41] Furfaro A. L. Piras S. Domenicotti C. Fenoglio D. De Luigi A. Salmona M. (2016). Role of Nrf2, HO-1 and GSH in neuroblastoma cell resistance to bortezomib. *Plos One* 11:e0152465. 10.1371/journal.pone.0152465 27023064 PMC4811586

[B42] Furfaro A. L. Ottonello S. Loi G. Cossu I. Piras S. Spagnolo F. (2019). HO-1 downregulation favors BRAFV600 melanoma cell death induced by Vemurafenib/PLX4032 and increases NK recognition. *Int. J. Cancer* 10.1002/ijc.32611 [Epub ahead of print]. 31376303

[B43] Gazzin S. Vitek L. Watchko J. Shapiro S. M. Tiribelli C. (2016). A novel perspective on the biology of bilirubin in health and disease. *Trends Mol. Med.* 22 758–768. 10.1016/j.molmed.2016.07.004 27515064

[B44] George E. M. Cockrell K. Aranay M. Csongradi E. Stec D. E. Granger J. P. (2011). Induction of heme oxygenase 1 attenuates placental ischemia-induced hypertension. *Hypertens Dallas Tex* 1979 941–948. 10.1161/HYPERTENSIONAHA.111.169755 21383306 PMC3085942

[B45] George E. M. Hosick P. A. Stec D. E. Granger J. P. (2013). Heme oxygenase inhibition increases blood pressure in pregnant rats. *Am. J. Hypertens* 26 924–930. 10.1093/ajh/hpt045 23553216 PMC3731822

[B46] Gill J. G. Piskounova E. Morrison S. J. (2016). Cancer, oxidative stress, and metastasis. *Cold Spring Harb. Symp. Quant. Biol.* 81 163–175. 10.1101/sqb.2016.81.030791 28082378

[B47] Gorrini C. Harris I. S. Mak T. W. (2013). Modulation of oxidative stress as an anticancer strategy. *Nat. Rev. Drug Discov.* 12 931–947. 10.1038/nrd4002 24287781

[B48] Grojean S. Koziel V. Vert P. Daval J. L. (2000). Bilirubin induces apoptosis via activation of NMDA receptors in developing rat brain neurons. *Exp. Neurol.* 166 334–341. 10.1006/exnr.2000.7518 11085898

[B49] Grosser N. Hemmerle A. Berndt G. Erdmann K. Hinkelmann U. Schürger S. (2004). The antioxidant defense protein heme oxygenase 1 is a novel target for statins in endothelial cells. *Free Radic. Biol. Med.* 37 2064–2071. 10.1016/j.freeradbiomed.2004.09.009 15544924

[B50] Ha J. G. Li L. Lee D. Na S. Ha K. S. Kim Y.-M. (2015). The effects of heme oxygenase by-products on the proliferation and invasion of HUVECs, HTR-8/SVneo cells, 3A(tPA 30-1) cells, and HESCs under varying oxygen concentrations. *Reprod. Sci. Thousand. Oaks Calif.* 22 1530–1538. 10.1177/1933719115589415 26040941

[B51] Habig W. H. Pabst M. J. Fleischner G. Gatmaitan Z. Arias I. M. Jakoby W. B. (1974). The identity of glutathione S-Transferase B with ligandin, a major binding protein of liver. *Proc. Natl. Acad. Sci. U.S.A.* 71 3879–3882. 10.1073/pnas.71.10.3879 4139704 PMC434288

[B52] Halilovic A. Patil K. A. Bellner L. Marrazzo G. Castellano K. Cullaro G. (2011). Knockdown of heme oxygenase-2 impairs corneal epithelial cell wound healing. *J. Cell Physiol.* 226 1732–1740. 10.1002/jcp.22502 21506105 PMC3078528

[B53] He M. Nitti M. Piras S. Furfaro A. L. Traverso N. Pronzato M. A. (2015). Heme oxygenase-1-derived bilirubin protects endothelial cells against high glucose-induced damage. *Free Radic. Biol. Med.* 89 91–98. 10.1016/j.freeradbiomed.2015.07.151 26391462

[B54] Hench P. S. (1938). Effect of jaundice on rheumatoid arthritis. *Br. Med. J.* 2 394–398. 10.1136/bmj.2.4050.394 20781677 PMC2210279

[B55] Hench P. S. (1949). Potential reversibility of rheumatoid arthritis. *Ann. Rheum. Dis.* 8 90–96. 10.1136/ard.8.2.90 18623811 PMC1030684

[B56] Higuchi S. Kabeya Y. Uchida J. Kato K. Tsukada N. (2018). Low bilirubin levels indicate a high risk of cerebral deep white matter lesions in apparently healthy subjects. *Sci. Rep.* 8:6473. 10.1038/s41598-018-24917-8 29691467 PMC5915409

[B57] Hinds T. D. Stec D. E. (2018). Bilirubin, a cardiometabolic signaling molecule. *Hypertens Dallas Tex* 72 788–795. 10.1161/HYPERTENSIONAHA.118.11130 30354722 PMC6205727

[B58] Hopkins P. N. Wu L. L. Hunt S. C. James B. C. Vincent G. M. Williams R. R. (1996). Higher serum bilirubin is associated with decreased risk for early familial coronary artery disease. *Arterioscler. Thromb. Vasc. Biol.* 16 250–255. 10.1161/01.atv.16.2.250 8620339

[B59] Hung S.-Y. Liou H.-C. Fu W.-M. (2010). The mechanism of heme oxygenase-1 action involved in the enhancement of neurotrophic factor expression. *Neuropharmacology* 58 321–329. 10.1016/j.neuropharm.2009.11.003 19925812

[B60] Ikeda Y. Hamano H. Satoh A. Horinouchi Y. Izawa-Ishizawa Y. Kihira Y. (2015). Bilirubin exerts pro-angiogenic property through Akt-eNOS-dependent pathway. *Hypertens Res. Off. J. Jpn. Soc. Hypertens* 38 733–740. 10.1038/hr.2015.74 26134126

[B61] Inoguchi T. Sasaki S. Kobayashi K. Takayanagi R. Yamada T. (2007). Relationship between gilbert syndrome and prevalence of vascular complications in patients with diabetes. *JAMA* 298 1398–1400. 10.1001/jama.298.12.1398-b 17895455

[B62] Jabarpour M. Siavashi V. Asadian S. Babaei H. Jafari S. M. Nassiri S. M. (2018). Hyperbilirubinemia-induced pro-angiogenic activity of infantile endothelial progenitor cells. *Microvasc Res* 118 49–56. 10.1016/j.mvr.2018.02.005 29476756

[B63] Jangi S. Otterbein L. Robson S. (2013). The molecular basis for the immunomodulatory activities of unconjugated bilirubin. *Int. J. Biochem. Cell Biol.* 45 2843–2851. 10.1016/j.biocel.2013.09.014 24144577

[B64] Jansen T. Daiber A. (2012). Direct antioxidant properties of bilirubin and biliverdin. is there a role for biliverdin reductase. *Front. Pharmacol.* 3:30. 10.3389/fphar.2012.00030 22438843 PMC3306014

[B65] Jansen T. Hortmann M. Oelze M. Opitz B. Steven S. Schell R. (2010). Conversion of biliverdin to bilirubin by biliverdin reductase contributes to endothelial cell protection by heme oxygenase-1-evidence for direct and indirect antioxidant actions of bilirubin. *J. Mol. Cell Cardiol.* 49 186–195. 10.1016/j.yjmcc.2010.04.011 20430037

[B66] Jazwa A. Stepniewski J. Zamykal M. Jagodzinska J. Meloni M. Emanueli C. (2013). Pre-emptive hypoxia-regulated HO-1 gene therapy improves post-ischaemic limb perfusion and tissue regeneration in mice. *Cardiovasc. Res.* 97 115–124. 10.1093/cvr/cvs284 23087099 PMC3527762

[B67] Jeong J. H. Kim H. G. Choi O. H. (2014). sildenafil inhibits advanced glycation end products-induced sFlt-1 release through upregulation of heme Oxygenase-1. *J. Menopausal. Med.* 20 57–68. 10.6118/jmm.2014.20.2.57 25371895 PMC4207003

[B68] Jirásková A. Novotný J. Novotný L. Vodicka P. Pardini B. Naccarati A. (2012). Association of serum bilirubin and promoter variations in HMOX1 and UGT1A1 genes with sporadic colorectal cancer. *Int. J. Cancer* 131 1549–1555. 10.1002/ijc.27412 22212955

[B69] Kamisako T. Kobayashi Y. Takeuchi K. Ishihara T. Higuchi K. Tanaka Y. (2000). Recent advances in bilirubin metabolism research: the molecular mechanism of hepatocyte bilirubin transport and its clinical relevance. *J. Gastroenterol.* 35 659–664. 10.1007/s005350070044 11023036

[B70] Kapitulnik J. (2004). Bilirubin: an endogenous product of heme degradation with both cytotoxic and cytoprotective properties. *Mol. Pharmacol.* 66 773–779. 10.1124/mol.104.002832 15269289

[B71] Kapitulnik J. Benaim C. Sasson S. (2012). Endothelial cells derived from the blood-brain barrier and islets of langerhans differ in their response to the effects of bilirubin on oxidative stress under hyperglycemic conditions. *Front. Pharmacol.* 3:131. 10.3389/fphar.2012.00131 22811666 PMC3396126

[B72] Kaur H. Hughes M. N. Green C. J. Naughton P. Foresti R. Motterlini R. (2003). Interaction of bilirubin and biliverdin with reactive nitrogen species. *FEBS Lett.* 543 113–119. 10.1016/S0014-5793(03)00420-4 12753916

[B73] Keshavan P. Deem T. L. Schwemberger S. J. Babcock G. F. Cook-Mills J. M. Zucker S. D. (2005). Unconjugated bilirubin inhibits VCAM-1-mediated transendothelial leukocyte migration. *J. Immunol. Baltim. Md.* 1950 3709–3718. 10.4049/jimmunol.174.6.3709 15749910

[B74] Kim J. Y. Lee D. Y. Kang S. Miao W. Kim H. Lee Y. (2017). Bilirubin nanoparticle preconditioning protects against hepatic ischemia-reperfusion injury. *Biomaterials* 133 1–10. 10.1016/j.biomaterials.2017.04.011 28414974

[B75] Kim S. Y. Kang H. T. Choi H. R. Park S. C. (2011). Biliverdin reductase A in the prevention of cellular senescence against oxidative stress. *Exp. Mol. Med.* 43 15–23. 10.3858/emm.2011.43.1.002 21099244 PMC3041934

[B76] Kim Y.-M. Pae H.-O. Park J. E. Lee Y. C. Woo J. M. Kim N.-H. (2011). Heme oxygenase in the regulation of vascular biology: from molecular mechanisms to therapeutic opportunities. *Antioxid. Redox Signal.* 14 137–167. 10.1089/ars.2010.3153 20624029 PMC2988629

[B77] Kishimoto Y. Kondo K. Momiyama Y. (2019). The protective role of heme oxygenase-1 in atherosclerotic diseases. *Int. J. Mol. Sci.* 20:3628. 10.3390/ijms20153628 31344980 PMC6695885

[B78] Konícková R. Vaòková K. Vaňková J. Váňová K. Muchová L. Subhanová I. (2014). Anti-cancer effects of blue-green alga spirulina platensis, a natural source of bilirubin-like tetrapyrrolic compounds. *Ann. Hepatol.* 13 273–283. 10.1016/s1665-2681(19)30891-9 24552870

[B79] Krönke G. Kadl A. Ikonomu E. Blüml S. Fürnkranz A. Sarembock I. J. (2007). Expression of heme oxygenase-1 in human vascular cells is regulated by peroxisome proliferator-activated receptors. *Arterioscler. Thromb. Vasc. Biol.* 27 1276–1282. 10.1161/ATVBAHA.107.142638 17413033

[B80] Kumagai A. Ando R. Miyatake H. Greimel P. Kobayashi T. Hirabayashi Y. (2013). A bilirubin-inducible fluorescent protein from eel muscle. *Cell* 153 1602–1611. 10.1016/j.cell.2013.05.038 23768684

[B81] Kundur A. R. Bulmer A. C. Singh I. (2014). Unconjugated bilirubin inhibits collagen induced platelet activation. *Platelets* 25 45–50. 10.3109/09537104.2013.764405 23406485

[B82] Kundur A. R. Santhakumar A. B. Bulmer A. C. Singh I. (2017). Mildly elevated unconjugated bilirubin is associated with reduced platelet activation-related thrombogenesis and inflammation in Gilbert’s syndrome. *Platelets* 28 779–785. 10.1080/09537104.2017.1280146 28300459

[B83] Kundur A. R. Singh I. Bulmer A. C. (2015). Bilirubin, platelet activation and heart disease: a missing link to cardiovascular protection in Gilbert’s syndrome? *Atherosclerosis* 239 73–84. 10.1016/j.atherosclerosis.2014.12.042 25576848

[B84] Lapenna D. Ciofani G. Pierdomenico S. D. Giamberardino M. A. Ucchino S. Davì G. (2018). Association of serum bilirubin with oxidant damage of human atherosclerotic plaques and the severity of atherosclerosis. *Clin. Exp. Med.* 18 119–124. 10.1007/s10238-017-0470-5 28948382

[B85] Lee Y. Lee S. Jon S. (2018). Biotinylated bilirubin nanoparticles as a tumor microenvironment-responsive drug delivery system for targeted cancer therapy. *Adv. Sci. Weinh. Baden- Wurtt. Ger.* 5:800017. 10.1002/advs.201800017 29938184 PMC6010876

[B86] Levi A. J. Gatmaitan Z. Arias I. M. (1969). Two hepatic cytoplasmic protein fractions, Y and Z, and their possible role in the hepatic uptake of bilirubin, sulfobromophthalein, and other anions. *J. Clin. Invest.* 48 2156–2167. 10.1172/JCI106182 4980931 PMC297469

[B87] Levytska K. Kingdom J. Baczyk D. Drewlo S. (2013). Heme oxygenase-1 in placental development and pathology. *Placenta* 34 291–298. 10.1016/j.placenta.2013.01.004 23403148

[B88] Li Y. Wang Q. Xu Q. Cai S. Zhou J. Ren B. (2014). Valsartan decreases neointimal hyperplasia in balloon-injured rat aortic arteries by upregulating HO-1 and inhibiting angiotensin II type 1 receptor. *Life Sci.* 110 70–76. 10.1016/j.lfs.2014.06.021 25014676

[B89] Li Z. Wang Y. Man R. Y. K. Vanhoutte P. M. (2013). Upregulation of heme oxygenase-1 potentiates EDH-type relaxations in the mesenteric artery of the spontaneously hypertensive rat. *Am. J. Physiol.Heart Circ. Physiol.* 305 H1471–H1483. 10.1152/ajpheart.00962.2012 24014672

[B90] Litwack G. Ketterer B. Arias I. M. (1971). Ligandin: a hepatic protein which binds steroids, bilirubin, carcinogens and a number of exogenous organic anions. *Nature* 234 466–467. 10.1038/234466a0 4944188

[B91] Liu J. Wang L. Tian X. Y. Liu L. Wong W. T. Zhang Y. (2015). Unconjugated bilirubin mediates heme oxygenase-1-induced vascular benefits in diabetic mice. *Diabetes* 64 1564–1575. 10.2337/db14-1391 25475440

[B92] Liu X.-M. Durante Z. E. Peyton K. J. Durante W. (2016). Heme oxygenase-1-derived bilirubin counteracts HIV protease inhibitor-mediated endothelial cell dysfunction. *Free Radic. Biol. Med.* 94 218–229. 10.1016/j.freeradbiomed.2016.03.003 26968795 PMC4844824

[B93] Liu Y. Li P. Lu J. Xiong W. Oger J. Tetzlaff W. (2008). Bilirubin possesses powerful immunomodulatory activity and suppresses experimental autoimmune encephalomyelitis. *J. Immunol. Baltim. Md.* 1950 1887–1897. 10.4049/jimmunol.181.3.1887 18641326

[B94] Loboda A. Jazwa A. Grochot-Przeczek A. Rutkowski A. J. Cisowski J. Agarwal A. (2008). Heme oxygenase-1 and the vascular bed: from molecular mechanisms to therapeutic opportunities. *Antioxid. Redox Signal.* 10 1767–1812. 10.1089/ars.2008.2043 18576916

[B95] Lundvig D. M. S. Immenschuh S. Wagener F. A. (2012). Heme oxygenase, inflammation, and fibrosis: the good, the bad, and the ugly? *Front. Pharmacol.* 3:81. 10.3389/fphar.2012.00081 22586396 PMC3345581

[B96] Maamoun H. Benameur T. Pintus G. Munusamy S. Agouni A. (2019). Crosstalk between oxidative stress and endoplasmic reticulum (ER) stress in endothelial dysfunction and aberrant angiogenesis associated with diabetes: a focus on the protective roles of heme oxygenase (HO)-1. *Front. Physiol.* 10:70. 10.3389/fphys.2019.00070 30804804 PMC6378556

[B97] Maamoun H. Zachariah M. McVey J. H. Green F. R. Agouni A. (2017). Heme oxygenase (HO)-1 induction prevents endoplasmic reticulum stress-mediated endothelial cell death and impaired angiogenic capacity. *Biochem. Pharmacol.* 127 46–59. 10.1016/j.bcp.2016.12.009 28012960

[B98] Maines M. D. (1988). Heme oxygenase: function, multiplicity, regulatory mechanisms, and clinical applications. *Faseb. J.* 2 2557–2568. 10.1096/fasebj.2.10.3290025 3290025

[B99] Maines M. D. Trakshel G. M. Kutty R. K. (1986). Characterization of two constitutive forms of rat liver microsomal heme oxygenase. Only one molecular species of the enzyme is inducible. *J. Biol. Chem.* 261 411–419. 3079757

[B100] Marengo B. Nitti M. Furfaro A. L. Colla R. Ciucis C. D. Marinari U. M. (2016). Redox homeostasis and cellular antioxidant systems: crucial players in cancer growth and therapy. *Oxid. Med. Cell Longev* 2016:6235641. 10.1155/2016/6235641 27418953 PMC4932173

[B101] Maruhashi T. Kihara Y. Higashi Y. (2019). Bilirubin and endothelial function. *J. Atheroscler. Thromb.* 26 688–696. 10.5551/jat.RV17035 31270300 PMC6711845

[B102] Maruhashi T. Soga J. Fujimura N. Idei N. Mikami S. Iwamoto Y. (2012). Hyperbilirubinemia, augmentation of endothelial function, and decrease in oxidative stress in Gilbert syndrome. *Circulation* 126 598–603. 10.1161/CIRCULATIONAHA.112.105775 22773454

[B103] Mason J. C. (2016). Cytoprotective pathways in the vascular endothelium. Do they represent a viable therapeutic target? *Vasc. Pharmacol.* 86 41–52. 10.1016/j.vph.2016.08.002 27520362

[B104] Mazzone G. L. Rigato I. Ostrow J. D. Bossi F. Bortoluzzi A. Sukowati C. H. (2009a). Bilirubin inhibits the TNFalpha-related induction of three endothelial adhesion molecules. *Biochem. Biophys. Res. Commun.* 386 338–344. 10.1016/j.bbrc.2009.06.029 19523446

[B105] Mazzone G. L. Rigato I. Ostrow J. D. Tiribelli C. (2009b). Bilirubin effect on endothelial adhesion molecules expression is mediated by the NF-kappaB signaling pathway. *Biosci. Trends.* 3 151–157. 20103840

[B106] McCarty M. F. (2007). “Iatrogenic Gilbert syndrome” –a strategy for reducing vascular and cancer risk by increasing plasma unconjugated bilirubin. *Med. Hypotheses* 69 974–994. 10.1016/j.mehy.2006.12.069 17825497

[B107] Minetti M. Mallozzi C. Di Stasi A. M. Pietraforte D. (1998). Bilirubin is an effective antioxidant of peroxynitrite-mediated protein oxidation in human blood plasma. *Arch. Biochem. Biophys.* 352 165–174. 10.1006/abbi.1998.0584 9587403

[B108] Mölzer C. Wallner M. Kern C. Tosevska A. Schwarz U. Zadnikar R. (2016). Features of an altered AMPK metabolic pathway in Gilbert’s syndrome, and its role in metabolic health. *Sci. Rep.* 6:30051. 10.1038/srep30051 27444220 PMC4956769

[B109] Moore K. J. Sheedy F. J. Fisher E. A. (2013). Macrophages in atherosclerosis: a dynamic balance. *Nat. Rev. Immunol.* 13 709–721. 10.1038/nri3520 23995626 PMC4357520

[B110] Morita T. Kourembanas S. (1995). Endothelial cell expression of vasoconstrictors and growth factors is regulated by smooth muscle cell-derived carbon monoxide. *J. Clin. Invest.* 96 2676–2682. 10.1172/JCI118334 8675634 PMC185974

[B111] Mortada I. (2017). Hyperbilirubinemia, hypertension, and CKD: the links. *Curr. Hypertens Rep.* 19:58. 10.1007/s11906-017-0756-8 28597405

[B112] Nam J. Lee Y. Yang Y. Jeong S. Kim W. Yoo J.-W. (2018). Is it worth expending energy to convert biliverdin into bilirubin? *Free Radic. Biol. Med.* 124 232–240. 10.1016/j.freeradbiomed.2018.06.010 29898414

[B113] NaveenKumar S. K. Thushara R. M. Sundaram M. S. Hemshekhar M. Paul M. Thirunavukkarasu C. (2015). Unconjugated bilirubin exerts pro-apoptotic effect on platelets via p38-MAPK activation. *Sci. Rep.* 5:15045. 10.1038/srep15045 26459859 PMC4602209

[B114] Neuzil J. Stocker R. (1993). Bilirubin attenuates radical-mediated damage to serum albumin. *FEBS Lett.* 331 281–284. 10.1016/0014-5793(93)80353-v 8375511

[B115] Nitti M. Piras S. Brondolo L. Marinari U. M. Pronzato M. A. Furfaro A. L. (2018). Heme oxygenase 1 in the nervous system: does it favor neuronal cell survival or induce neurodegeneration? *Int. J. Mol. Sci.* 19:E2260. 10.3390/ijms19082260 30071692 PMC6121636

[B116] Nitti M. Piras S. Marinari U. M. Moretta L. Pronzato M. A. Furfaro A. L. (2017). HO-1 induction in cancer progression: a matter of cell adaptation. *Antioxid. Basel. Switz.* 6 E29. 10.3390/antiox6020029 28475131 PMC5488009

[B117] Novotný L. Vítek L. (2003). Inverse relationship between serum bilirubin and atherosclerosis in men: a meta-analysis of published studies. *Exp. Biol. Med. Maywood. N. J.* 228 568–571. 10.1177/15353702-0322805-29 12709588

[B118] Ollinger R. Kogler P. Troppmair J. Hermann M. Wurm M. Drasche A. (2007). Bilirubin inhibits tumor cell growth via activation of ERK. *Cell Cycle Georget Tex* 6:3078–3085. 10.4161/cc.6.24.5022 18073533

[B119] Ostrow J. D. Pascolo L. Shapiro S. M. Tiribelli C. (2003). New concepts in bilirubin encephalopathy. *Eur. J. Clin. Invest.* 33 988–997. 10.1046/j.1365-2362.2003.01261.x 14636303

[B120] Otterbein L. E. Foresti R. Motterlini R. (2016). Heme oxygenase-1 and carbon monoxide in the heart: the balancing act between danger signaling and pro-survival. *Circ. Res.* 118 1940–1959. 10.1161/CIRCRESAHA.116.306588 27283533 PMC4905590

[B121] Palmela I. Sasaki H. Cardoso F. L. Moutinho M. Kim K. S. Brites D. (2012). Time-dependent dual effects of high levels of unconjugated bilirubin on the human blood-brain barrier lining. *Front. Cell Neurosci.* 6:22. 10.3389/fncel.2012.00022 22590454 PMC3349234

[B122] Park J.-S. Nam E. Lee H.-K. Lim M. H. Rhee H.-W. (2016). In cellulo mapping of subcellular localized bilirubin. *ACS Chem. Biol.* 11 2177–2185. 10.1021/acschembio.6b00017 27232847

[B123] Peng F. Deng X. Yu Y. Chen X. Shen L. Zhong X. (2011). Serum bilirubin concentrations and multiple sclerosis. *J. Clin. Neurosci. Off. J. Neurosurg. Soc. Australas.* 18 1355–1359. 10.1016/j.jocn.2011.02.023 21782448

[B124] Peng F. Yang Y. Liu J. Jiang Y. Zhu C. Deng X. (2012). Low antioxidant status of serum uric acid, bilirubin and albumin in patients with neuromyelitis optica. *Eur. J. Neurol.* 19 277–283. 10.1111/j.1468-1331.2011.03488.x 21801283

[B125] Pennell E. N. Wagner K.-H. Mosawy S. Bulmer A. C. (2019). Acute bilirubin ditaurate exposure attenuates ex vivo platelet reactive oxygen species production, granule exocytosis and activation. *Redox. Biol.* 26:101250. 10.1016/j.redox.2019.101250 31226648 PMC6586953

[B126] Peterson S. J. Rubinstein R. Faroqui M. Raza A. Boumaza I. Zhang Y. (2019). Positive effects of heme oxygenase upregulation on adiposity and vascular dysfunction: gene targeting vs. Pharmacologic Therapy. *Int. J. Mol. Sci.* 20:E2514. 10.3390/ijms20102514 31121826 PMC6566770

[B127] Pflueger A. Croatt A. J. Peterson T. E. Smith L. A. d’Uscio L. V. Katusic Z. S. (2005). The hyperbilirubinemic gunn rat is resistant to the pressor effects of angiotensin II. *Am. J. Physiol. Renal. Physiol.* 288 F552–F558. 10.1152/ajprenal.00278.2004 15536166

[B128] Piras S. Furfaro A. L. Brondolo L. Passalacqua M. Marinari U. M. Pronzato M. A. (2017). Differentiation impairs Bach1 dependent HO-1 activation and increases sensitivity to oxidative stress in SH-SY5Y neuroblastoma cells. *Sci. Rep.* 7:7568. 10.1038/s41598-017-08095-7 28790431 PMC5548785

[B129] Qaisiya M. Coda Zabetta C. D. Bellarosa C. Tiribelli C. (2014). Bilirubin mediated oxidative stress involves antioxidant response activation via Nrf2 pathway. *Cell Signal.* 26 512–520. 10.1016/j.cellsig.2013.11.029 24308969

[B130] Quan S. Kaminski P. M. Yang L. Morita T. Inaba M. Ikehara S. (2004). Heme oxygenase-1 prevents superoxide anion-associated endothelial cell sloughing in diabetic rats. *Biochem. Biophys. Res. Commun.* 315 509–516. 10.1016/j.bbrc.2004.01.086 14766238

[B131] Ram M. Singh V. Kumar D. Kumawat S. Gopalakrishnan A. Lingaraju M. C. (2014). Antioxidant potential of bilirubin-accelerated wound healing in streptozotocin-induced diabetic rats. *Naunyn Schmiedebergs Arch. Pharmacol.* 387 955–961. 10.1007/s00210-014-1011-3 24969350

[B132] Ram M. Singh V. Kumawat S. Kant V. Tandan S. K. Kumar D. (2016). Bilirubin modulated cytokines, growth factors and angiogenesis to improve cutaneous wound healing process in diabetic rats. *Int. Immunopharmacol.* 30 137–149. 10.1016/j.intimp.2015.11.037 26679676

[B133] Ramma W. Ahmed A. (2014). Therapeutic potential of statins and the induction of heme oxygenase-1 in preeclampsia. *J. Reprod. Immunol.* 10 153–160. 10.1016/j.jri.2013.12.120 24503248 PMC4003533

[B134] Rochette L. Zeller M. Cottin Y. Vergely C. (2018). Redox functions of heme oxygenase-1 and biliverdin reductase in diabetes. *Trends Endocrinol. Metab.* 29 74–85. 10.1016/j.tem.2017.11.005 29249571

[B135] Rodrigues C. M. Sola S. Silva R. F. Brites D. (2002). Aging confers different sensitivity to the neurotoxic properties of unconjugated bilirubin. *Pediatr. Res.* 51 112–118. 10.1203/00006450-200201000-00020 11756649

[B136] Ryter S. W. Otterbein L. E. Morse D. Choi A. M. (2002). Heme oxygenase/carbon monoxide signaling pathways: regulation and functional significance. *Mol. Cell Biochem.* 23 249–263. 10.1007/978-1-4615-1087-1_29 PMC7101540 12162441

[B137] Sedlak T. W. Saleh M. Higginson D. S. Paul B. D. Juluri K. R. Snyder S. H. (2009). Bilirubin and glutathione have complementary antioxidant and cytoprotective roles. *Proc. Natl. Acad. Sci. U.S.A* 106 5171–5176. 10.1073/pnas.0813132106 19286972 PMC2664041

[B138] Sekioka R. Tanaka M. Nishimura T. Itoh H. (2015). Serum total bilirubin concentration is negatively associated with increasing severity of retinopathy in patients with type 2 diabetes mellitus. *J. Diabetes Complications* 29 218–221. 10.1016/j.jdiacomp.2014.12.002 25536865

[B139] Seta F. Bellner L. Rezzani R. Regan R. F. Dunn M. W. Abraham N. G. (2006). Heme oxygenase-2 is a critical determinant for execution of an acute inflammatory and reparative response. *Am. J. Pathol.* 169 1612–1623. 10.2353/ajpath.2006.060555 17071585 PMC1780218

[B140] Seyed Khoei N. Grindel A. Wallner M. Mölzer C. Doberer D. Marculescu R. (2018). Mild hyperbilirubinaemia as an endogenous mitigator of overweight and obesity: Implications for improved metabolic health. *Atherosclerosis* 269 306–311. 10.1016/j.atherosclerosis.2017.12.021 29279144

[B141] Shan H. Li T. Zhang L. Yang R. Li Y. Zhang M. (2019). Heme oxygenase-1 prevents heart against myocardial infarction by attenuating ischemic injury-induced cardiomyocytes senescence. *EBioMedicine* 39 59–68. 10.1016/j.ebiom.2018.11.056 30527623 PMC6355645

[B142] Shapiro S. M. (2003). Bilirubin toxicity in the developing nervous system. *Pediatr. Neurol.* 29 410–421. 10.1016/j.pediatrneurol.2003.09.011 14684236

[B143] Silva R. F. Rodrigues C. M. Brites D. (2002). Rat cultured neuronal and glial cells respond differently to toxicity of unconjugated bilirubin. *Pediatr. Res.* 51 535–541. 10.1203/00006450-200204000-00022 11919342

[B144] Siow R. C. Sato H. Mann G. E. (1999). Heme oxygenase-carbon monoxide signalling pathway in atherosclerosis: anti-atherogenic actions of bilirubin and carbon monoxide? *Cardiovasc. Res.* 41 385–394. 10.1016/s0008-6363(98)00278-8 10341838

[B145] Stec D. E. John K. Trabbic C. J. Luniwal A. Hankins M. W. Baum J. (2016). Bilirubin binding to PPARα inhibits lipid accumulation. *PloS One* 11:e0153427. 10.1371/journal.pone.0153427 27071062 PMC4829185

[B146] Stec D. E. Storm M. V. Pruett B. E. Gousset M. U. (2013). Antihypertensive actions of moderate hyperbilirubinemia: role of superoxide inhibition. *Am. J. Hypertens* 26 918–923. 10.1093/ajh/hpt038 23482378 PMC3731819

[B147] Stocker R. Yamamoto Y. McDonagh A. F. Glazer A. N. Ames B. N. (1987). Bilirubin is an antioxidant of possible physiological importance. *Science* 235 1043–1046. 10.1126/science.3029864 3029864

[B148] Šuk J. Jašprová J. Biedermann D. Petrásková L. Valentová K. Křen V. (2019). Isolated silymarin flavonoids increase systemic and hepatic bilirubin concentrations and lower lipoperoxidation in mice. *Oxid. Med. Cell Longev* 2019:6026902. 10.1155/2019/6026902 30891115 PMC6390243

[B149] Sundararaghavan V. L. Binepal S. Stec D. E. Sindhwani P. Hinds T. D. (2018). Bilirubin, a new therapeutic for kidney transplant? *Transplant. Rev. Orlando. Fla* 32 234–240. 10.1016/j.trre.2018.06.003 29983261 PMC6535229

[B150] Takeda T. A. Mu A. Tai T. T. Kitajima S. Taketani S. (2015). Continuous de novo biosynthesis of haem and its rapid turnover to bilirubin are necessary for cytoprotection against cell damage. *Sci. Rep.* 5:10488. 10.1038/srep10488 25990790 PMC4438432

[B151] Thakkar M. Edelenbos J. Doré S. (2019). Bilirubin and ischemic stroke: rendering the current paradigm to better understand the protective effects of bilirubin. *Mol. Neurobiol.* 56 5483–5496. 10.1007/s12035-018-1440-y 30612336

[B152] Tosevska A. Moelzer C. Wallner M. Janosec M. Schwarz U. Kern C. (2016). Longer telomeres in chronic, moderate, unconjugated hyperbilirubinaemia: insights from a human study on Gilbert’s syndrome. *Sci. Rep.* 6:22300. 10.1038/srep22300 26926838 PMC4772088

[B153] Traverso N. Ricciarelli R. Nitti M. Marengo B. Furfaro A. L. Pronzato M. A. (2013). Role of glutathione in cancer progression and chemoresistance. *Oxid. Med Cell Longev* 2013:972913. 10.1155/2013/972913 23766865 PMC3673338

[B154] Vanková K. Marková I. Jašprová J. Dvořák A. Subhanová I. Zelenka J. (2018). Chlorophyll-mediated changes in the redox status of pancreatic cancer cells are associated with its anticancer effects. *Oxid. Med. Cell Longev* 2018:4069167. 10.1155/2018/4069167 30057678 PMC6051000

[B155] Vera T. Granger J. P. Stec D. E. (2009). Inhibition of bilirubin metabolism induces moderate hyperbilirubinemia and attenuates ANG II-dependent hypertension in mice. *Am. J. Physiol. Regul. Integr. Comp. Physiol.* 297 R738–R743. 10.1152/ajpregu.90889.2008 19571206 PMC2739796

[B156] Vera T. Stec D. E. (2010). Moderate hyperbilirubinemia improves renal hemodynamics in ANG II-dependent hypertension. *Am. J. Physiol. Regul. Integr. Comp. Physiol.* 299 R1044–R1049. 10.1152/ajpregu.00316.2010 20668235 PMC2957382

[B157] Viallard C. Larrivée B. (2017). Tumor angiogenesis and vascular normalization: alternative therapeutic targets. *Angiogenesis* 20 409–426. 10.1007/s10456-017-9562-9 28660302

[B158] Vítek L. (2012). The role of bilirubin in diabetes, metabolic syndrome, and cardiovascular diseases. *Front. Pharmacol.* 3:55. 10.3389/fphar.2012.00055 22493581 PMC3318228

[B159] Vítek L. (2017). Bilirubin and atherosclerotic diseases. *Physiol. Res.* 66 S11–S20. 28379026 10.33549/physiolres.933581

[B160] Vitek L. Bellarosa C. Tiribelli C. (2019a). Induction of mild hyperbilirubinemia: hype or real therapeutic opportunity? *Clin. Pharmacol. Ther.* 106 568–575. 10.1002/cpt.1341 30588615

[B161] Vitek L. Hubacek J. A. Pajak A. Doryńska A. Kozela M. Eremiasova L. (2019b). Association between plasma bilirubin and mortality. *Ann. Hepatol.* 18 379–385. 10.1016/j.aohep.2019.02.001 31054979

[B162] Vítek L. Jirsa M. Brodanová M. Kalab M. Marecek Z. Danzig V. (2002). Gilbert syndrome and ischemic heart disease: a protective effect of elevated bilirubin levels. *Atherosclerosis* 160 449–456. 10.1016/s0021-9150(01)00601-3 11849670

[B163] Vítek L. Muchová L. Janèová E. Pešičková S. Tegzová D. Peterová V. (2010). Association of systemic lupus erythematosus with low serum bilirubin levels. *Scand. J. Rheumatol.* 39 480–484. 10.3109/03009741003742748 20604673

[B164] Vogel M. E. Idelman G. Konaniah E. S. Zucker S. D. (2017). Bilirubin prevents atherosclerotic lesion formation in low-density lipoprotein receptor-deficient mice by inhibiting endothelial VCAM-1 and ICAM-1 signaling. *J. Am. Heart Assoc.* 6:e004820. 10.1161/JAHA.116.004820 28365565 PMC5532999

[B165] Vogel M. E. Zucker S. D. (2016). Bilirubin acts as an endogenous regulator of inflammation by disrupting adhesion molecule-mediated leukocyte migration. *Inflamm. Cell Signal.* 3:e1178. 10.14800/ics.1178 26925435 PMC4768809

[B166] Wagner K.-H. Wallner M. Mölzer C. Gazzin S. Bulmer A. C. Tiribelli C. (2015). Looking to the horizon: the role of bilirubin in the development and prevention of age-related chronic diseases. *Clin. Sci. Lond. Engl.* 129 1–25. 10.1042/CS20140566 25881719

[B167] Wallner M. Antl N. Rittmannsberger B. Schreidl S. Najafi K. Müllner E. (2013). Anti-genotoxic potential of bilirubin in vivo: damage to DNA in hyperbilirubinemic human and animal models. *Cancer Prev. Res. Phila. Pa* 6 1056–1063. 10.1158/1940-6207.CAPR-13-0125 23983086

[B168] Wallner M. Blassnigg S. M. Marisch K. Pappenheim M. T. Müllner E. Mölzer C. (2012). Effects of unconjugated bilirubin on chromosomal damage in individuals with Gilbert’s syndrome measured with the micronucleus cytome assay. *Mutagenesis* 27 731–735. 10.1093/mutage/ges039 22874647

[B169] Wang D. Tosevska A. Heiß E. H. Ladurner A. Mölzer C. Wallner M. (2017). Bilirubin decreases macrophage cholesterol efflux and ATP-binding cassette transporter a1 protein expression. *J. Am. Heart Assoc.* 6:e005520. 10.1161/JAHA.117.005520 28455345 PMC5524097

[B170] Wang Q. Doerschuk C. M. (2000). Neutrophil-induced changes in the biomechanical properties of endothelial cells: roles of ICAM-1 and reactive oxygen species. *J. Immunol. Baltim. Md.* 1950 6487–6494. 10.4049/jimmunol.164.12.6487 10843706

[B171] Wang Q. Doerschuk C. M. (2002). The signaling pathways induced by neutrophil-endothelial cell adhesion. *Antioxid. Redox Signal.* 4 39–47. 10.1089/152308602753625843 11970842

[B172] Wang W. Lo A. C. Y. (2018). Diabetic retinopathy: pathophysiology and treatments. *Int. J. Mol. Sci.* 19 E1816. 10.3390/ijms19061816 29925789 PMC6032159

[B173] Wu B. J. Chen K. Barter P. J. Rye K.-A. (2012). Niacin inhibits vascular inflammation via the induction of heme oxygenase-1. *Circulation* 125 150–158. 10.1161/CIRCULATIONAHA.111.053108 22095827

[B174] Wu T. W. Fung K. P. Yang C. C. (1994). Unconjugated bilirubin inhibits the oxidation of human low density lipoprotein better than Trolox. *Life Sci.* 54 477–481. 8201841 10.1016/0024-3205(94)90140-6

[B175] Xiao Y. Xia J. Wu S. Lv Z. Huang S. Huang H. (2018). curcumin inhibits acute vascular inflammation through the activation of heme Oxygenase-1. *Oxid. Med. Cell Longev* 2018:3295807. 10.1155/2018/3295807 30327711 PMC6171216

[B176] Yang Q. He G.-W. Underwood M. J. Yu C.-M. (2016). Cellular and molecular mechanisms of endothelial ischemia/reperfusion injury: perspectives and implications for postischemic myocardial protection. *Am. J. Transl. Res.* 8 765–777. 27158368 PMC4846925

[B177] Yasuda M. Kiyohara Y. Wang J. J. Arakawa S. Yonemoto K. Doi Y. (2011). High serum bilirubin levels and diabetic retinopathy: the Hisayama Study. *Ophthalmology* 118 1423–1428. 10.1016/j.ophtha.2010.12.009 21600659

[B178] Young J. L. Libby P. Schönbeck U. (2002). Cytokines in the pathogenesis of atherosclerosis. *Thromb. Haemost.* 88 554–567. 10.1055/s-0037-1613256 12362224

[B179] Yu M. Su D. Yang Y. Qin L. Hu C. Liu R. (2019). D-T7 peptide-modified pegylated bilirubin nanoparticles loaded with cediranib and paclitaxel for antiangiogenesis and chemotherapy of glioma. *ACS Appl. Mater. Interfaces* 11 176–186. 10.1021/acsami.8b16219 30525386

[B180] Zelenka J. Dvořák A. Alán L. Zadinová M. Haluzík M. Vítek L. (2016). Hyperbilirubinemia protects against aging-associated inflammation and metabolic deterioration. *Oxid Med. Cell Longev* 2016:6190609. 10.1155/2016/6190609 27547293 PMC4983390

[B181] Zelenka J. Muchova L. Zelenkova M. Vanova K. Vreman H. J. Wong R. J. (2012). Intracellular accumulation of bilirubin as a defense mechanism against increased oxidative stress. *Biochimie* 94 1821–1827. 10.1016/j.biochi.2012.04.026 22580386

[B182] Zhao H. Ozen M. Wong R. J. Stevenson D. K. (2015). Heme oxygenase-1 in pregnancy and cancer: similarities in cellular invasion, cytoprotection, angiogenesis, and immunomodulation. *Front. Pharmacol.* 5:295. 10.3389/fphar.2014.00295 25642189 PMC4294126

[B183] Zhao H. Wong R. J. Doyle T. C. Nayak N. Vreman H. J. Contag C. H. (2008). Regulation of maternal and fetal hemodynamics by heme oxygenase in mice. *Biol. Reprod.* 78 744–751. 10.1095/biolreprod.107.064899 18094356

[B184] Ziberna L. Martelanc M. Franko M. Passamonti S. (2016). Bilirubin is an endogenous antioxidant in human vascular endothelial cells. *Sci. Rep.* 6:29240. 10.1038/srep29240 27381978 PMC4933905

[B185] Zou Z.-Y. Liu J. Chang C. Li J.-J. Luo J. Jin Y. (2019). Biliverdin administration regulates the microRNA-mRNA expressional network associated with neuroprotection in cerebral ischemia reperfusion injury in rats. *Int. J. Mol. Med.* 43 1356–1372. 10.3892/ijmm.2019.4064 30664169 PMC6365090

[B186] Zucker S. D. Horn P. S. Sherman K. E. (2004). Serum bilirubin levels in the U.S. population: gender effect and inverse correlation with colorectal cancer. *Hepatol. Baltim. Md.* 40 827–835. 10.1002/hep.20407 15382174

[B187] Zucker S. D. Vogel M. E. Kindel T. L. Smith D. L. H. Idelman G. Avissar U. (2015). Bilirubin prevents acute DSS-induced colitis by inhibiting leukocyte infiltration and suppressing upregulation of inducible nitric oxide synthase. *Am. J. Physiol. Gastrointest. Liver Physiol.* 309 G841–G854. 10.1152/ajpgi.00149.2014 26381705 PMC4652140

